# Proteomic analysis of human prostate cancer PC-3M-1E8 cells and PC-3M-2B4 cells of same origin but with different metastatic potential

**DOI:** 10.1371/journal.pone.0206139

**Published:** 2018-10-31

**Authors:** Shujiang Zhang, Chengcheng Zheng, Shunheng Yao, Zhonghui Wang, Li Xu, Rongfu Yang, Xiang Meng, Jianhui Wu, Li Zhou, Zuyue Sun

**Affiliations:** 1 School of Pharmacy, Fudan University, Shanghai, P. R., China; 2 Department of Pharmacology and Toxicology, Shanghai Institute of Planned Parenthood Research, Shanghai, P. R., China; 3 Key Laboratory of Contraceptive Drugs and Devices of National Population and Family Planning Commission of China, Shanghai Institute of Planned Parenthood Research, Shanghai, P. R., China; 4 Institute of Reproduction and Development, Fudan University, Shanghai, P. R., China; Southern Illinois University School of Medicine, UNITED STATES

## Abstract

Prostate cancer (PCa) is the second most frequently diagnosed cancer and the fifth leading cause of death from cancer in men worldwide. Increased understanding of the prostate cancer metastasis mechanisms will help identify more efficient intervention strategies to prevent or treat this deadly disease in the future. To identify the candidate proteins that contribute to metastasis of PCa, isobaric tags for relative and absolute quantitation (iTRAQ)-based proteomic analysis was performed to explore differentially expressed proteins between two homologous human prostate cancer cell lines including highly-metastatic PC-3M-1E8 cell line and poorly-metastatic PC-3M-2B4 cell line. Here, a total of 58 proteins were identified to be significantly differentially expressed between PC-3M-1E8 and PC-3M-2B4 cells, which were further verified using real-time quantitative PCR and western blot analysis. The bioinformatic analysis suggested that the differentially expressed proteins, like MMP1 and FHL1, may contribute to the higher metastatic ability of PC-3M-1E8 cells than PC-3M-2B4 cells. In addition, functional analyses proved MMP1’s positive effect on the higher metastatic ability of PC-3M-1E8 cells than PC-3M-2B4 cells. These findings provided a unique resource to specifically reveal the complex molecular regulatory mechanisms underlying the progression of prostate cancer from poorly-metastatic to highly-metastatic stage.

## Introduction

Prostate cancer (PCa) is the second most common cancer and the fifth most fatal cancer among men worldwide [1]. In the United States, 161,360 new prostate cancer cases and 26,730 deaths are projected to occur in 2017, making it the most common cancer and the third leading cause of cancer death in men [2]. With its morbidity and mortality rates increasing rapidly in the past decade, it became the most common urologic malignancy in China as a result of the increased aging population, gradual implementation of prostate-specific antigen (PSA) screening, improved biopsy techniques, the impact of an increasingly westernized lifestyle, etc [3]. Although the localized PCa can be well controlled through watchful waiting, radical prostatectomy or radiotherapy, it remains incurable at the stage of lethal metastatic PCa and its mechanisms are not well elucidated. Molecular mechanisms research directed toward largely unknown PCa metastasis will help us discover novel therapeutic targets and improve intervention strategies for treatment of this deadly disease.

*In vitro* cell-based models that closely mimic the clinical condition in patients are crucial to understand the pathogenesis of prostate cancer and develop novel therapeutic agents. *In vitro* model experiments are more flexible than xenografts, with high control over environmental factors and unlimited sample amounts, although xenografts are similar to the *in vivo* environment of the patient more closely. Moreover, *in vitro* cell lines contribute to identify the pathogenesis of certain kind of cells and eliminate the influence of epithelial/stromal interactions and vascularization. Homologous cell line model system and resource consists of some cell lines, for example, androgen sensitive prostate cancer cell line LNCaP and its sublines androgen-insensitive JHU-LNCaPSM [4], androgen-independent LNCaP-CS10 [5], and androgen suppressed LNCS [6], which have the same genetic origin but represent different phases of clinical PCa, from androgen sensitive growth, through androgen independence, to androgen suppression, so clarifying their unique genetic differences are valuable for prostate cancer progression disparity research; for another example, the human prostate epithelial cancer cell line PC-3M [7] and its sublines, highly-metastatic potential cell line PC-3M-1E8 cells and poorly-metastatic potential cell line PC-3M-2B4 cells [8], these two cell lines that derived from the same lineage are valuable cell-based models to study the molecular mechanisms of prostate cancer metastasis *in vitro* and *in vivo*, so they are important tools for investigating the biology of the prostate cancer progression from poorly-metastatic to highly-metastatic phase. In addition, poorly-metastatic PC-3M-2B4 cells and their homologous highly-metastatic subline PC-3M-1E8 cells provide a unique *in vitro* model system and resource for PCa disparity research.

Even though the molecular pathogenesis of prostate cancer metastases has been intensely studied for over 70 years, there is still much to be understood. Comparing highly-metastatic PC-3M-1E8 cells with their homologous poorly-metastatic PC-3M-2B4 cells may help identify important pathways in the pathogenesis of prostate cancer metastases. To identify the candidate proteins that contribute to metastasis of prostate cancer, we performed an isobaric tags for relative and absolute quantitation (iTRAQ)-based quantitative proteomic analysis to screen differentially expressed proteins between two paired homologous human prostate cancer cell lines including highly-metastatic PC-3M-1E8 cell line and poorly-metastatic PC-3M-2B4 cell line. The differentially expressed proteins were validated by real-time quantitative PCR (qRT-PCR) and western blot analysis. Based on bioinformatic analysis, MMP1 and FHL1 are potentially functional proteins associated with differences of PC-3M-1E8 and PC-3M-2B4 cell lines in metastatic ability. In addition, functional analyses demonstrated that silencing MMP1 remarkably weakened the *in vitro* migratory and invasive capabilities of the PC-3M-1E8 cells, suggesting that MMP1 may be proved to be an ideal therapeutic target for overcoming prostate cancer metastasis.

## Materials and methods

### Cell lines and culture conditions

Human prostate cancer cell lines PC-3M-1E8 and PC-3M-2B4 cells were purchased from Cell Resource Center, IBMS, CAMS/PUMC. All cell lines were maintained as monolayer cultures in RPMI-1640 medium (Corning) supplemented with 10% fetal bovine serum (FBS; Hyclone) and penicillin-streptomycin (100 IU/ml and 100 μg/ml, respectively; Beyotime) in a humidified atmosphere of 5% CO_2_ at 37°C and were typically split at 70~80% confluency using 0.05% trypsin-0.02% ethylenediamine-tetraacetic acid (EDTA; Corning).

### PC-3M-1E8 and PC-3M-2B4 cell lines authentication

Nineteen short tandem repeat (STR) loci plus the gender determining locus, amelogenin, were simultaneously co-amplified from PC-3M-1E8, PC-3M-2B4 and PC-3M cells using the EX20 kit (AGCU) according to the manufacture's protocol. The cell line samples were processed using the Prism 3500 Genetic Analyzer (Applied Biosystems) and the data were analyzed using GeneMapper ID-X v1.2 software (Applied Biosystems) by the Genetic Testing Biotechnology Corporation (Suzhou). Appropriate positive and negative controls were run and confirmed for each sample submitted. The resulting profile showed the assigned allele values corresponding to the number of repeat units identified for each locus. Cell lines were authenticated using STR analysis as described in ANSI Standard (ASN-0002) by the ATCC Standards Development Organization (SDO) in 2012.

### Western blot analysis

Whole cell protein lysate was extracted using RIPA lysis buffer (Solarbio) according to protocols provided by the manufacturer. Protein concentrations were determined by BCA Protein Assay Kit (Beyotime). Equal amounts of proteins (10 or 20 μg) were separated by SDS-PAGE and the resolved proteins were transferred onto a PVDF membrane (Millipore). After being blocked with 5% nonfat powdered milk in TBST [20 mM Tris–HCl (pH 7.5), 150 mM NaCl, 0.05% Tween-20] for 1 hr at room temperature, membranes were incubated with primary antibody for 1 hr at room temperature or overnight at 4°C. Primary antibodies Cytokeratin 8 (CK8), CK5, Vimentin, hepatocyte growth factor α (HGFα), and four-and-a-half LIM domains 1 (FHL1) were obtained from Santa Cruz Biotechnology, matrix metalloproteinase 1 (MMP1) and CK19 from Abcam, housekeeping protein glyceraldehyde 3-phosphate dehydrogenase (GAPDH) from Cell Signaling Technology. Details regarding the primary antibodies were provided in the S1 Table. Total protein loading was assessed using GAPDH antibody. Bands were visualized using horse-radish peroxidase (HRP) conjugated secondary anti-mouse (Santa Cruz) or anti-rabbit (Cell Signaling Technology) antibodies in conjunction with chemiluminescent HRP substrate (Merck Millipore) via Tanon-5200.

### Cell migration and invasion assays

Cell migration assays were performed using 6.5 mm Transwell with 8.0 μm pore polycarbonate membrane insert (Corning) as previously described [9] with some modifications. In brief, cells (1.0 × 10^5^) in 200 μl serum-free RPMI-1640 medium were loaded into the top chambers, and 600 μl RPMI-1640 media containing 10% FBS was added into the lower chamber as a chemoattractant. After incubation for forty-eight hours at 37°C in 5% CO_2_, the non-migratory cells on the upper surface were removed by a cotton swab and the cells that had migrated to the other side of the membrane were fixed with 4% paraformaldehyde for 20 minutes and stained with Wright-Giemsa. Values for migration were obtained by counting cells in 10 fields (2 × (centre + 4 quadrants)) per membrane (× 20 objective) and averaged for three wells. For cell invasion assays, cells (1.0 × 10^5^) were suspended in 200 μl serum-free RPMI-1640 and seeded on the matrigel-coated membrane in each insert of biocoat matrigel invasion chambers with 8.0 μm PET membrane (Corning). Forty-eight hours later, the cells that had invaded the matrigel and moved to the other side of the membrane were fixed, stained and counted as in the migration assay. The experiment was repeated three times independently.

### Cell scratch assay

The scratch healing assay was used to investigate cell migration *in vitro*. Assays were performed as the instructions of the Culture–Insert (ibidi). Briefly, applied 70 μl cell suspension (4 × 10^5^ cells/ml) into each well and allowed to grow to a confluent monolayer at 37°C and 5% CO_2_ for 24 hours. Gently removed the Culture–Insert using a sterile tweezer, then cells were washed with PBS and 2 ml RPMI-1640 culture medium containing 1% FBS was added. Following incubation of the plate for 0, 6, 12 and 24 h at 37°C, at least two random pictures were captured for each well using an inverted microscope at 100 × magnification. Cell migration distance was quantitatively analyzed using the Image-Pro Plus software, and cell migration distance = the initial scratch width value—the present scratch width value. Experiments were performed by triplicate and results were represented as mean ± Standard Deviation (SD). The experiment was repeated three times independently.

### Protein extraction and denaturation

Cells were washed with cold phosphate-buffered saline (PBS) twice, harvested by scraping, then centrifuged at 1000 g for 5 min at 4°C. Cell pellets were resuspended in SDT lysis buffer containing 4% SDS (Bio‐Rad), 100 mM Tris-HCl (Sigma), 1 mM DTT (Bio‐Rad), pH 7.6 and sonicated on ice, then boiled for 15 min. After centrifuged at 14000 g for 40 min at 4°C to remove cell debris, the supernatants were collected and quantified with the BCA Protein Assay Kit (Beyotime). The supernatants were stored at -80°C until use. Proteins were denatured according to the filter-aided sample preparation (FASP) procedure with a few adjustments [10]. 200 μg proteins of each sample were incorporated into 30 μl SDT buffer (4% SDS, 100 mM DTT, 150 mM Tris-HCl, pH 8.0). The detergent, DTT and other low-molecular-weight components were removed by repeated ultrafiltration (Microcon units, 10 kD) using UA buffer (8 M Urea, 150 mM Tris-HCl, pH 8.0). Next, 100 μl iodoacetamide (100 mM in UA buffer) was added to block reduced cysteine residues, then the samples were incubated for 30 min in darkness. The filters were washed with 100 μl UA buffer three times and then washed with 100 μl dissolution buffer (DS buffer; AB SCIEX) twice.

### Protein digestion and iTRAQ labeling

The protein suspensions were digested with 4 μg trypsin (Promega) in 40 μl DS buffer overnight at 37°C, and the tryptic peptides were collected as a filtrate. The peptide content was measured by UV light spectral density at 280 nm. 100 μg tryptic peptide mixture of each sample was labeled with an iTRAQ Reagent - 8plex kit (Applied Biosystems) according to the manufacturer’s instructions. The two cell lines were labeled as follows: PC-3M-1E8-1-113, PC-3M-1E8-2-114 and PC-3M-1E8-3-115 (biological replications), PC-3M-2B4-1-116, PC-3M-2B4-2-117 and PC-3M-2B4-3-118 (biological replications). Three biological replications for both PC-3M-1E8 cells and PC-3M-2B4 cells were performed with three different passage cells.

### Peptide fractionation with strong cation exchange (SCX) chromatography

iTRAQ labeled peptides were fractionated with SCX chromatography through the AKTA Purifier System (GE Healthcare). The dried peptide mixture was reconstituted and acidified with 2 ml buffer A [10 mM KH_2_PO_4_ in 25% acetonitrile (CAN), pH 3.0] and then loaded onto a polysulfoethyl 4.6 x 100 mm column (5 μm, 200 Å, PolyLC Inc). The peptides were eluted at 1 ml/min with gradients of 0–8% buffer B [500 mM KCl, and 10 mM KH_2_PO_4_ in 25% CAN, pH 3.0] for 22 min, 8–52% buffer B during 22–47 min, 52–100% buffer B during 47–50 min, 100% buffer B during 50–58 min, and 0% buffer B after 58 min. The elution was monitored by absorbance at 214 nm and the fractions were collected every minute. The collected fractions were desalted on C18 Cartridges (Sigma) and concentrated by vacuum centrifugation.

### Liquid chromatography (LC)—Mass spectrometer (MS)/MS analysis

LC-MS/MS analysis was performed on a Q Exactive mass spectrometer (Thermo Scientific) coupled to Easy nLC liquid chromatograph (Thermo Scientific) for 60 min. For Nano-LC-MS/MS analysis, the peptide mixture (5 μg) was loaded onto a reverse phase trap column (Thermo Scientific Acclaim PepMap100, 100 μm x 2 cm, nanoViper C18) connected to the C18-reversed phase analytical column (Thermo Scientific Easy Column, 10 cm long, 75 μm inner diameter, 3 μm resin) in buffer A (0.1% formic acid) and separated with buffer B (84% acetonitrile, 0.1% formic acid) in linear gradients at 300 nl/min: 0–35% buffer B for 50 min, 35–100% buffer B for 5 min, and 100% buffer B for 5 min. The mass spectrometer was operated in positive ion mode. The MS data was collected with a data-dependent top10 method by dynamically acquiring the most abundant precursor ions from the survey scan (300–1800 m/z) of higher energy collisional dissociation (HCD) fragmentation. Automatic gain control (AGC) target was set at 3e6, maximum injection time at 10 ms, and dynamic exclusion duration at 40.0 s. Survey scans were acquired at a resolution of 70,000 at 200 m/z, resolution for HCD spectra at 17,500 at 200 m/z, and isolation width at 2 m/z. Normalized collision energy was defined as 30 eV and the underfill ratio as 0.1%. The instrument was run in peptide recognition mode.

### Protein identification and quantification

All raw files were analyzed using the MASCOT engine (Matrix Science; version 2.2) embedded into Proteome Discoverer 1.4 (Thermo Electron) against the Uniprot Human database (151619 sequences, downloaded on February 22, 2016). For protein identification, the following parameters were used: Peptide Mass Tolerance: ±20 ppm, Fragment Mass Tolerance: ±0.1 Da, Enzyme: trypsin, Mass Values: Monoisotopic, Max Missed Cleavages: 2, Fixed Modifications: Carbamidomethyl (C), iTRAQ 8plex (N-term), iTRAQ 8plex (K), Variable Modifications: Oxidation (M), iTRAQ 8plex (K), Database Pattern: Target-Decoy, and Peptide FDR ≤ 0.01. The relative quantitative analysis of the proteins in the samples based on the ratios of iTRAQ reporter ions from all unique peptides was performed using Proteome Discoverer (version 1.4). The relative peak intensities of the iTRAQ reporter ions released in each MS/MS spectra were used, and the PC-3M-2B4 sample was employed as a reference for calculating the iTRAQ ratios of all reporter ions. Then, the final ratios obtained from the relative protein quantifications were normalized based on the median protein ratio. The protein ratios are calculated with the median of the unique peptides of the protein.

### Bioinformatics analysis of the differentially abundant proteins

The sequence data of the selected differentially abundant proteins were retrieved from the UniProtKB database (Release 201607) in batches and FASTA format. The retrieved sequences were locally searched against Swiss-Prot database (human) using the NCBI BLAST + client software (ncbi-blast-2.2.28+-win32.exe) to find homologue sequences and their functional annotations were transferred to the identified sequences. The top 10 blast hits with E-value< = 1*e*-3 for each query sequence were retrieved and loaded into Blast2GO Command Line (Version: go_201504.obo; www.geneontology.org) for Gene Ontology (GO) mapping and annotation [11, 12]. The sequences without BLAST hits and the unannotated ones were then selected for InterProScan against EBI databases to retrieve their functional annotations and merge the InterProScan GO terms to the annotation set [13]. Following annotation and annotation augmentation, the studied proteins were searched against Kyoto Encyclopedia of Genes and Genomes (KEGG) genes (human) to retrieve their KEGG orthology identifications and were subsequently mapped to pathways in KEGG [14]. The hierarchical cluster analysis was carried out using the Cluster Software 3.0, and the result was compiled using Java Trewview. To construct a protein-protein interaction (PPI) network, we matched the significantly differently expressed proteins with regulatory data in IntAct (http://www.ebi.ac.uk/intact/main.xhtml) database and the resulting network was visualized with Cytoscape 3.2.1.

### Transcriptional validation by real-time quantitative PCR (qRT-PCR) analysis

Total RNA was extracted using TRIzol reagent (Thermo Fisher Scientific) and quantifed using a Multiskcan GO (Thermo Fisher Scientific). The RNA was reverse transcribed into the frst-strand cDNA using the RevertAid First Strand cDNA Synthesis Kit (Thermo Fisher Scientific). mRNA levels were determined by qRT-PCR using SYBR Premix Ex Taq (Tli RNaseH Plus; Takara) according to the manufacturer’s protocol using gene-specific primers (S2 Table) on the StepOne Real-Time PCR System (Applied Biosystems). GAPDH was employed as the internal reference gene to normalize the expression data. All gene relative transcription levels were calculated according to the 2^-ΔΔCt (cycle threshold)^ method. Each sample was tested in triplicate and three independent experiments were used to quantify relative gene transcription.

### Cell transfection

GV248-Lentivirus carrying small interference RNA (siRNA) targeting human MMP1 and non-targeting negative control (NC) were constructed (GeneChem). The viruses were transfected into cells using enhanced infection solution (GeneChem) in the presence of 5 μg/ml polybrene (GeneChem) and the medium was changed 8 hr later. The clones stably knocked down MMP1 were identified and confirmed using qRT-PCR and western blot analysis, as described previously. The siRNA sequences were as follows, MMP1: 5’-TTGTGGCTTATGGATTCAT-3’; the non-targeting negative control, 5’- TTCTCCGAACGTGTCACGT -3’.

### Statistical analysis

The data are presented as means ± SD. Student’s t-test (two-tailed) was performed to determine the significance of differences between PC-3M-1E8 and PC-3M-2B4 cells with *P* < 0.05 considered statistically significant.

## Results

### Genetic confirmation of PC-3M origin

To confirm the genetic lineage identity of PC-3M-1E8 and PC-3M-2B4 cell lines as derivatives of PC-3M cells, we compared the DNA fingerprinting patterns of PC-3M-1E8 and PC-3M-2B4 cells with that of the PC-3M cells at complete short tandem repeat (STR) profile (19 STR loci plus the gender determining locus, Amelogenin). This technique discriminates unrelated human cell lines uniquely through examining highly polymorphic STR DNA loci. Amelogenin is a DNA locus used for gender identification. STR data were analyzed following the ANSI Standard (ASN-0002) in 2012 of the ATCC Standards Development Organization (SDO), which revealed that PC-3M-1E8 and PC-3M-2B4 cells had an identical STR profile for allele 1 and 2 at all 19 STR markers and Amelogenin locus (only had X chromosome) to PC-3M cells (Table 1) and confirmed that PC-3M-1E8 and PC-3M-2B4 cell lines are sublines of PC-3M cells.

**Table 1:** STR Analysis for Genetic confirmation of PC-3M origin.

Target Loci	PC-3M-1E8	PC-3M-2B4	PC-3M	Alleles Match
Amelogenin	X	X	X	100%
D3S1358	16	16	16	
D13S317	11	11	11	
D7S820	8 11	8 11	8 11	
D16S539	11	11	11	
Penta E	10 17	10 17	10 17	
TPOX	8 9	8 9	8 9	
TH01	6 7	6 7	6 7	
D2S1338	18 20	18 20	18 20	
CSF1PO	11	11	11	
Penta D	9	9	9	
D19S433	14	14	14	
vWA	17	17	17	
D21S11	29 31.2	29 31.2	29 31.2	
D18S51	14 15	14 15	14 15	
D6S1043	14 18	14 18	14 18	
D8S1179	13	13	13	
D5S818	13	13	13	
D12S391	21	21	21	
FGA	24 25	24 25	24 25	

### Expression of cytodifferentiation markers

**Fig 1:**
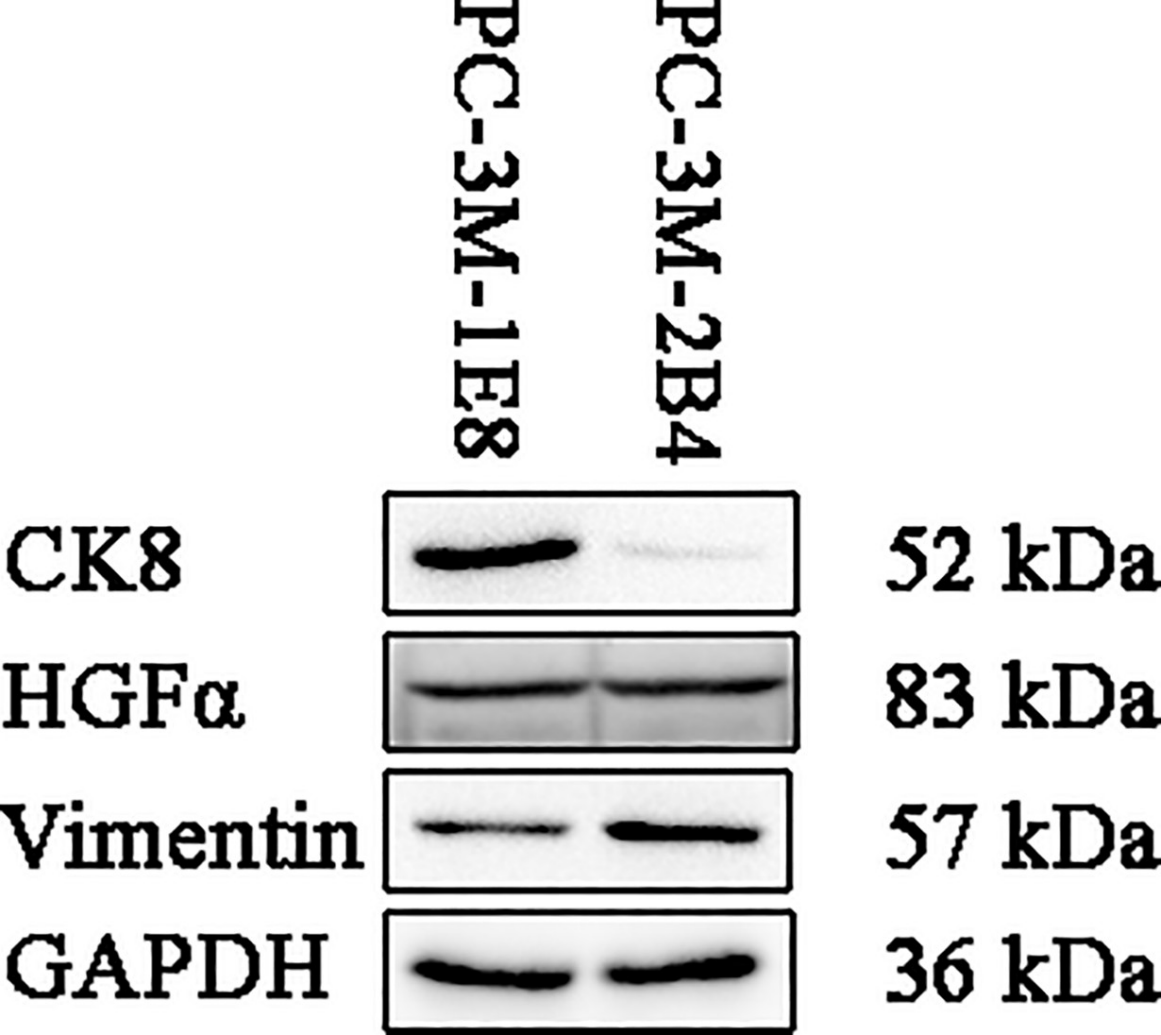
Expression of cytodifferentiation markers in PC-3M-1E8 and PC-3M-2B4 cells. Western blot analysis was performed on whole cell lysate using antibodies against epithelial (CK8), stromal (HGFα), and fibroblast (vimentin) markers. GAPDH was used as protein loading control. Full-length gels are presented in S1 Fig. Experiments were repeated three times independently.

A panel of epithelial (CK8), basal (CK5), fibroblast (vimentin), and stromal (HGFα) markers were determined by western blot analysis on whole cell lysates to confirm the cell types of PC-3M-1E8 and PC-3M-2B4 cell lines. Western blot analysis revealed that PC-3M-1E8 and PC-3M-2B4 cells expressed prostatic luminal secretory epithelial cell markers CK8, fibroblast marker vimentin and the stromal marker HGFα (Fig 1), while they were negative for the basal cell marker CK5 (data not shown). PC-3M-1E8 cells were strongly positive for CK8, but moderately expressed vimentin, in contrast, PC-3M-2B4 cells were strongly positive for vimentin, but moderately expressed CK8 (Fig 1). Cytodifferentiation markers were reproducible when they are tested at three different passages. Taken together, expression of secretory epithelial markers CK8 is in accordance with the epithelial origin for both PC-3M-1E8 and PC-3M-2B4 cell lines, in addition, expression of vimentin and HGFα by the PC-3M-1E8 and PC-3M-2B4 cells and higher expression level in vimentin but lower in CK8 by the PC-3M-2B4 cells than the PC-3M-1E8 cells suggest that they have differentiated towards mesenchymal cells and PC-3M-2B4 cells differentiated more than PC-3M-1E8 cells.

### PC-3M-1E8 cells have more metastatic potential *in vitro* than PC-3M-2B4 cells

**Fig 2:**
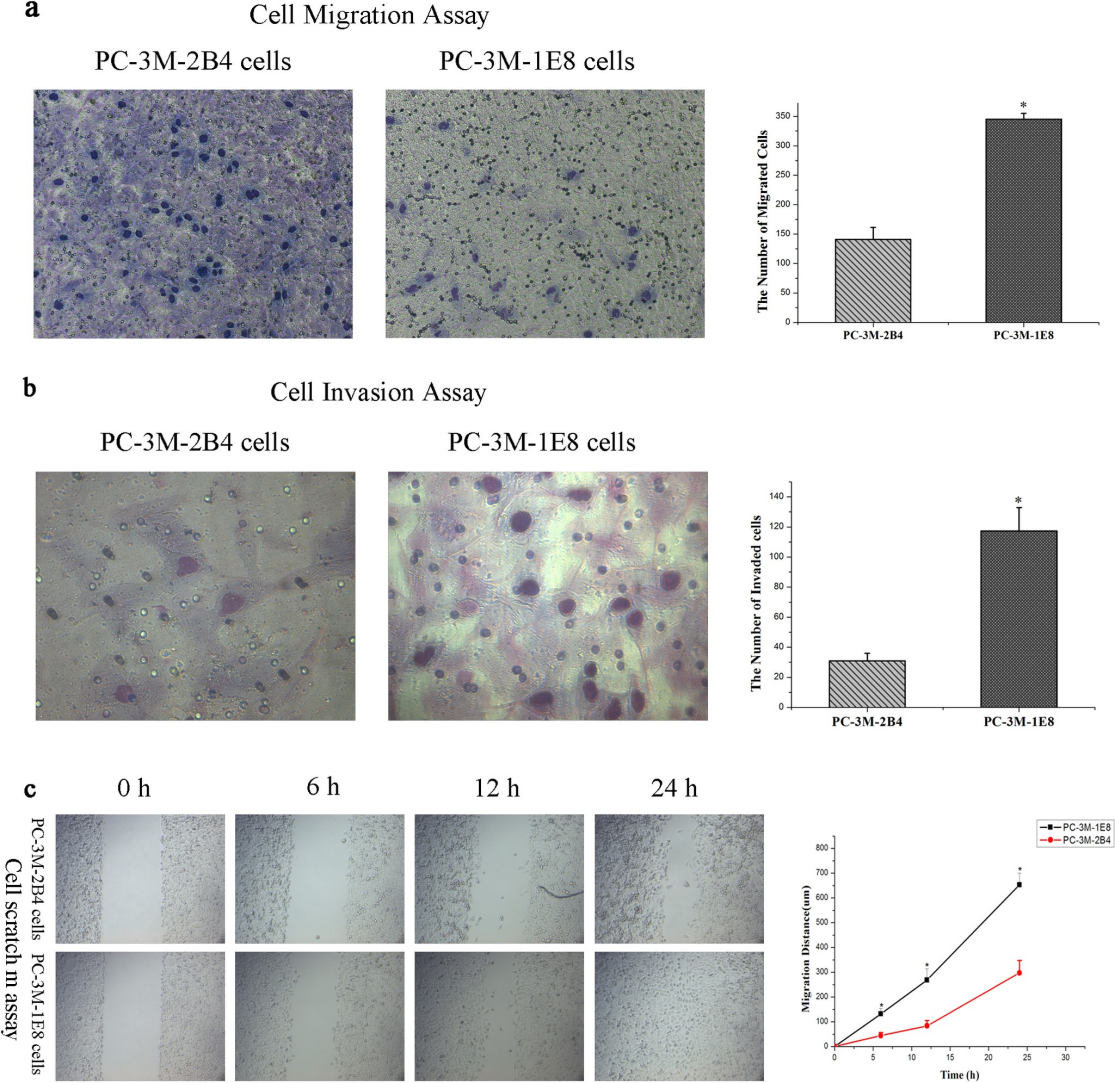
*In vitro* migration and invasion potential of PC-3M-1E8 cells and PC-3M-2B4 cells. (a) Representative photomicrographs of PC-3M-1E8 cells and PC-3M-2B4 cells are presented at 200 × magnification for migration assay using a microscope (left). Cells migrated were counted from ten fields (2 × (centre + 4 quadrants)). Bar graphs present relative differences in quantity of migrated cells for PC-3M-1E8 cells and PC-3M-2B4 cells (right). (b) Representative photomicrographs of PC-3M-1E8 cells and PC-3M-2B4 cells are presented at 400 × magnification for invasion assay using a microscope (left). Cells invaded were counted from ten fields (2×(centre+4 quadrants)). Bar graphs present relative differences in quantity of invaded cells for PC-3M-1E8 cells and PC-3M-2B4 cells (right) (c) Cell migration evaluated by scratch wound healing assay. Representative images of PC-3M-1E8 cells and PC-3M-2B4 cells migrating into the wounded area at 0, 6, 12 and 24 hr after wound formation are shown (left), and the wound surface areas were measured with Image-Pro Plus software. Line graph presents change in closure of wounds for PC-3M-1E8 cells and PC-3M-2B4 cells (right). The data represents mean ± SD of 3 repeats. There was a significant difference between PC-3M-1E8 cells and PC-3M-2B4 cells (Student’s t-test; *P<0.05).

The cell migration assay lacking an extracellular matrix barrier scores the simple migratory abilities of cancer cells *in vitro*, whereas the cell invasion assay measures the capabilities of cancer cells to degrade and invade Matrigel matrix *in vitro* that mimics the *in vivo* matrix barrier. In order to confirm PC-3M-1E8 cells have greater metastatic potential than PC-3M-2B4 cells as per the literature [8], we examined the cell migratory and invasive abilities of them. As shown in Fig 2A and 2B, the number of cells that migrated and invaded across 8 μm diameter pores over 48 h was significantly (*P* < 0.05) increased in PC-3M-1E8 cells in comparison with PC-3M-2B4 cells, indicating that the cellular migratory and invasive abilities *in vitro* of PC-3M-1E8 cells were stronger than that of PC-3M-2B4 cells. Cell motility is important for tumor cell invasion and metastasis [15]. *In vitro* cell scratch assays that preserve cell-cell interactions and are able to mimic migration of cells *in vivo* [16] were carried out to confirm whether PC-3M-1E8 cells migrated faster than PC-3M-2B4 cells. PC-3M-1E8 cells showed statistically significant faster in scratch closure than PC-3M-2B4 cells as early as 6 hr (Fig 2C). The scratch was completely closed by PC-3M-1E8 cells at 24 hr, whereas, PC-3M-2B4 cells showed much open spaces of the scratch at this same time point (*P* < 0.05), suggesting that PC-3M-1E8 cells migrated faster *in vitro* than PC-3M-2B4 cells. These results verified PC-3M-1E8 cells have significantly greater metastatic ability *in vitro* compared with PC-3M-2B4 cells.

### Differential protein abundances of PC-3M-1E8 cells and PC-3M-2B4 cells

**Fig 3:**
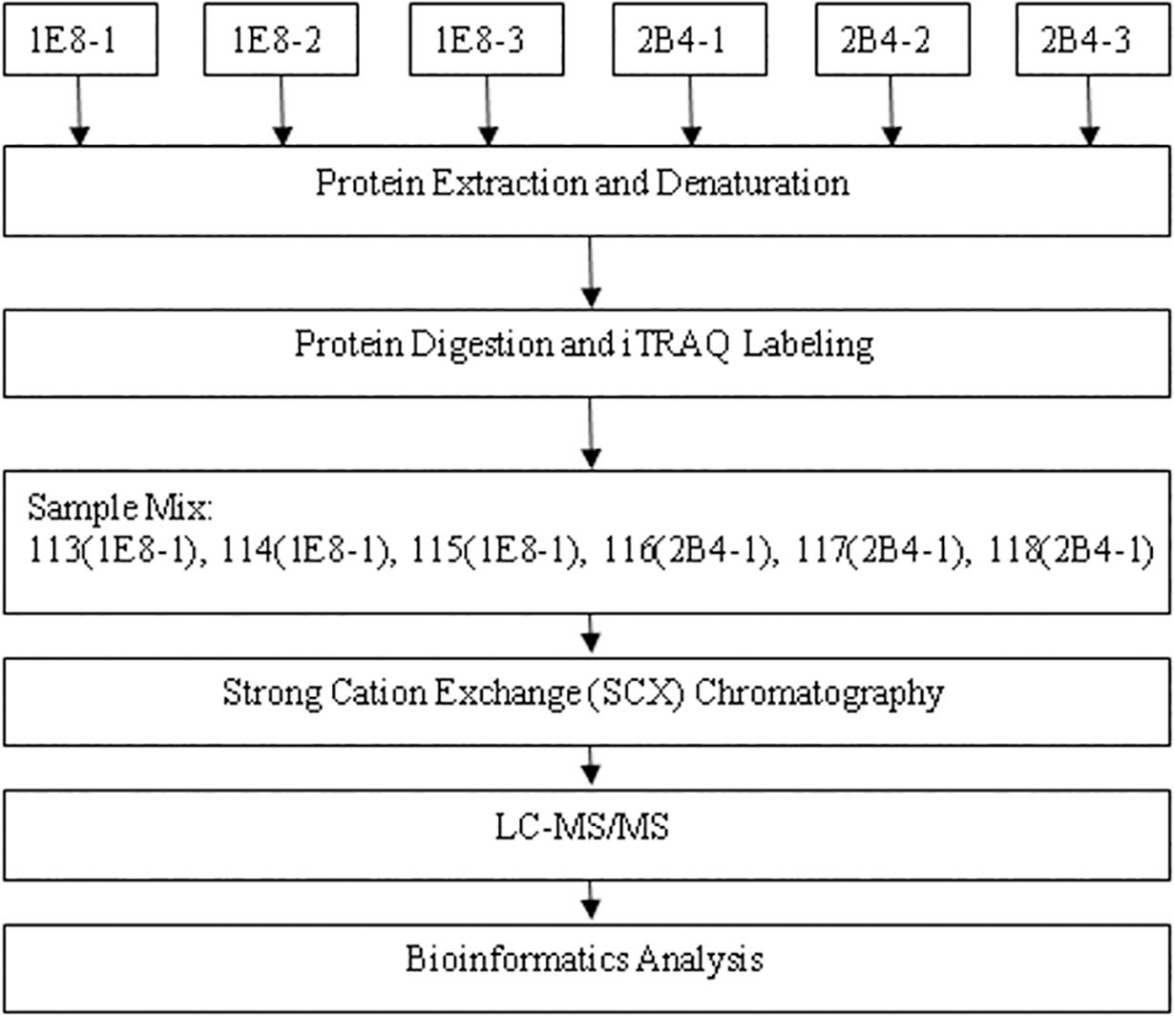
Schematics of the workflow for the iTRAQ proteomic analysis. The numbers including 113, 114, 115, 116, 117 and 118 are different iTRAQ labels.

**Fig 4:**
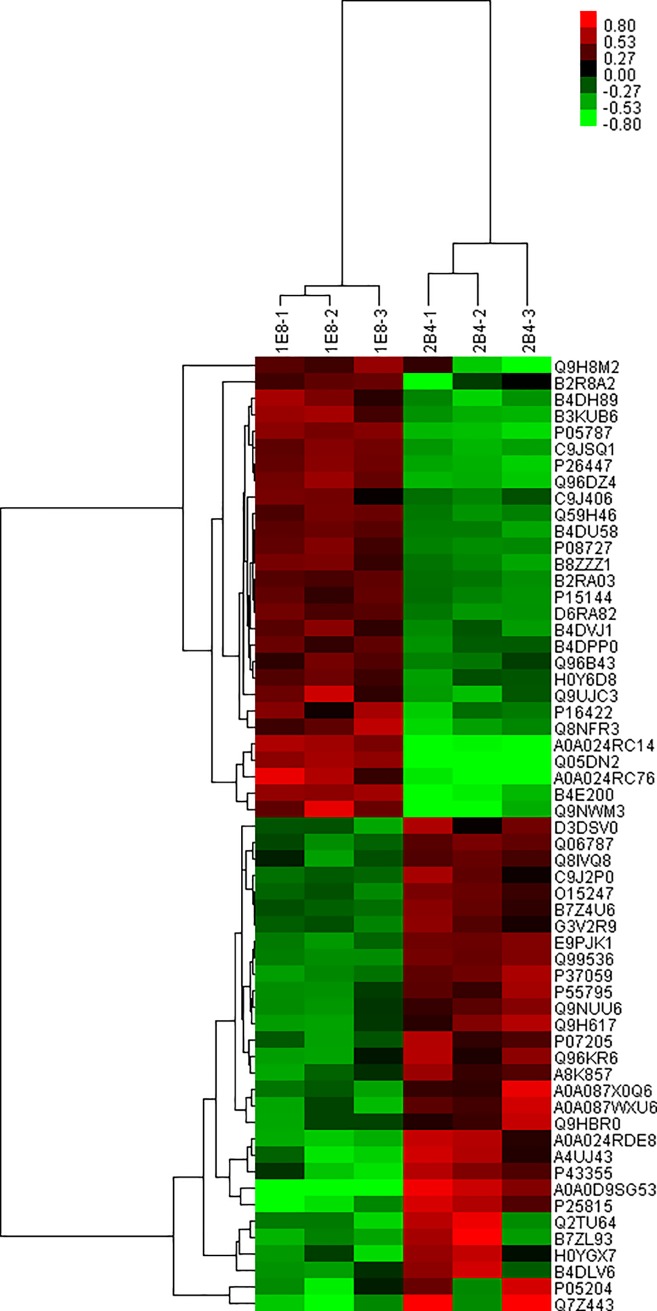
Clustering analysis of differentially expressed proteins. The color scale bar in the right of hierarchical clustering analysis indicates that proteins activated in PC-3M-1E8 compared with PC-3M-2B4 are depicted in red and proteins repressed are depicted in green.

We performed iTRAQ analysis on the total cellular proteins of PC-3M-1E8 cells and PC-3M-2B4 cells as shown in Fig 3. Same amounts of proteins from PC-3M-1E8 cells and PC-3M-2B4 cells were digested by trypsin and the resulting peptide mixture was labeled with iTRAQ reagents (113, 114, 115 for PC-3M-1E8 cells; 116, 117, 118 for PC-3M-2B4 cells). The iTRAQ labeled peptides were fractionated by SCX chromatography, then the eluted fractions were analyzed by LC-MS/MS. The recognized peptides were then searched against the Uniprot Human database. A total of 37,627 peptides (S3 Table) matching 6054 proteins (≥ 1 unique peptide; S4 Table) were identified from the two cell lines analysis. The protein fold changes of PC-3M-1E8 cells and PC-3M-2B4 cells were determined by the ratios of their iTRAQ reporter ions. 58 proteins (28 up-regulated and 30 down-regulated; S5 Table and Fig 4) were identified according to a screening criteria ≥± 1.5-fold change and a *P* ≤ 0.05. The top five up-regulated proteins in the proteomics analysis are interferon-induced protein with tetratricopeptide repeats 2 (IFIT2), interferon stimulated exonuclease gene 20kDa (ISG20), melanoma antigen family A, 10 (MAGEA10), CUE domain-containing protein 1 (CUEDC1) and peptidyl-prolyl cis-trans isomerase C (PPIC). The top 5 down-regulated proteins are four and a half LIM domains protein 1 (FHL1), protein S100-P, polycystic kidney disease protein 1-like 3 (PKD1L3), PDZ and LIM domain 5, isoform CRA_c (PDLIM5) and glutathione S-transferase.

### GO annotation of the differentially abundant proteins

**Fig 5:**
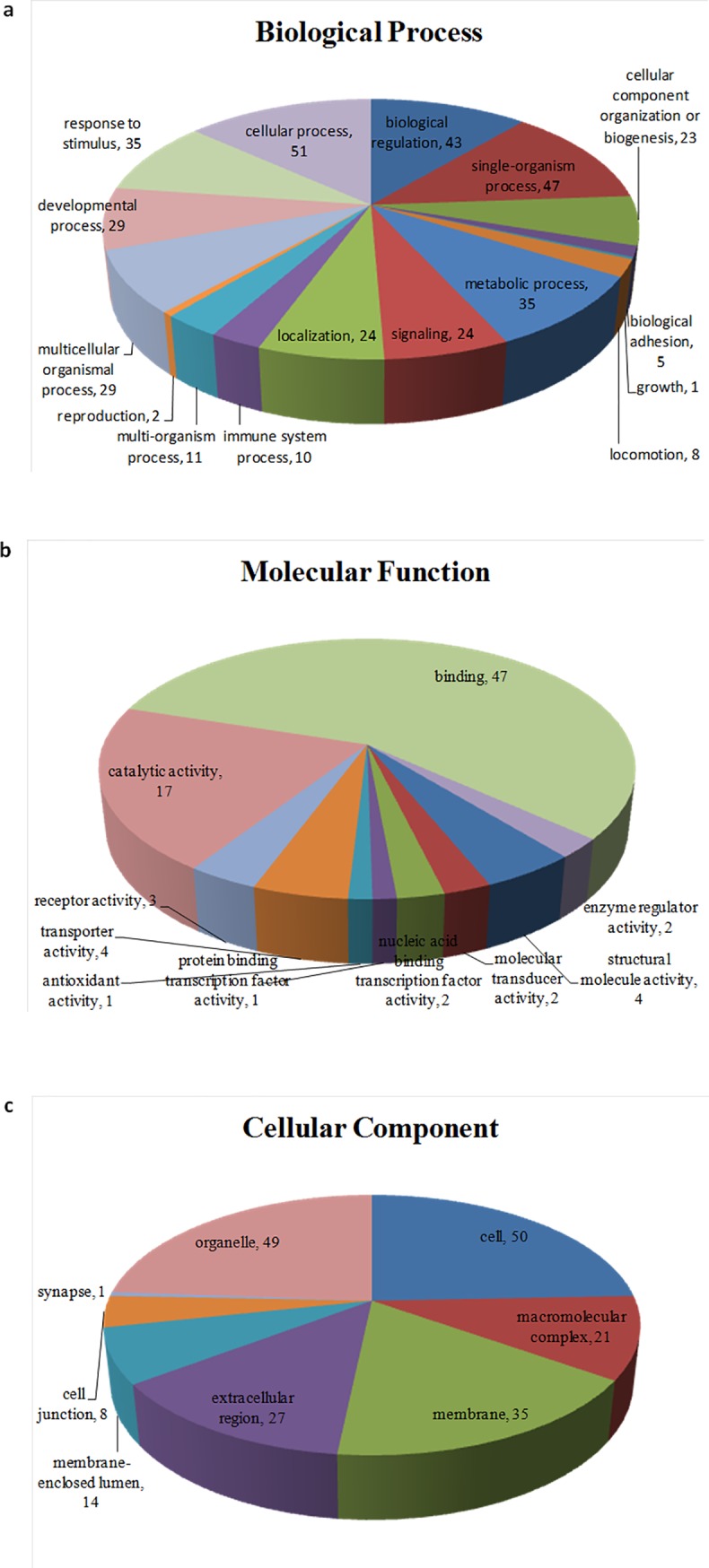
**Distribution of GO functional categories in biological process (a), molecular functions (b), and cellular component (c).** All data are presented on the basis of GO level 2 terms. Numbers refer to assigned proteins in each category.

**Fig 6:**
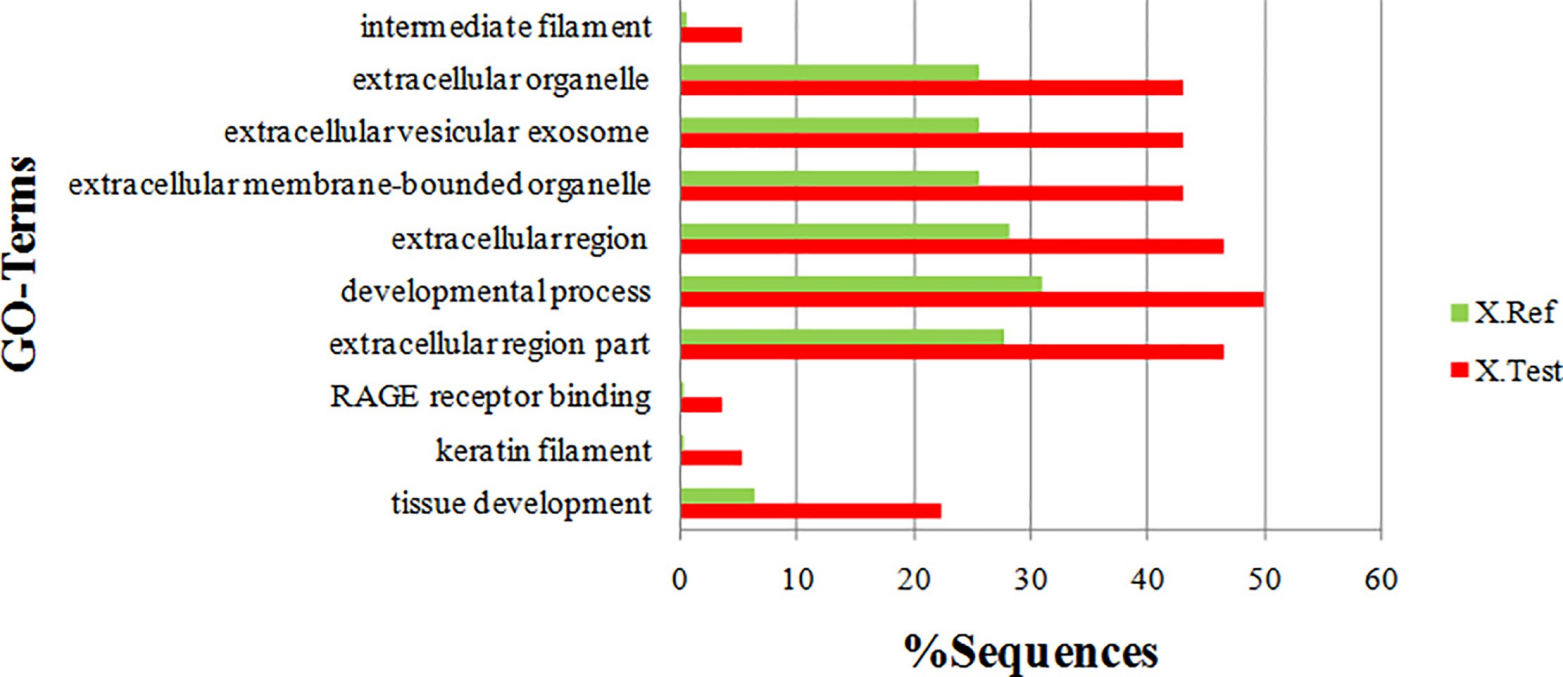
GO enrichment analysies of differentially expressed proteins. All data are presented on the basis of GO level 2 terms. Green and red bars represent the GO term corresponding proteins’ proportions in the collection of all qualitative proteins and the GO term corresponding differentially expressed proteins’ proportions in their collection, respectively. Statistical analysis was performed using Fisher’s exact test; they are significant different from each other, *P*< 0.01.

All the 58 proteins identified via iTRAQ were subsequently classified according to GO annotations. The GO database is an internationally standardized functional classification system to comprehensively describe characteristics of different genes and their products in terms of their involvement in three main categories: biological process, cellular component, and molecular function [12]. According to GO annotations, all 58 proteins identified differentially expressed in PC-3M-1E8 cells compared with PC-3M-2B4 cells were annotated to 817 GO entries and each protein was assigned at least one term. GO terms were applied to classify proteins into three main categories, including biological processes, molecular functions, and cellular components: as summarized in Fig 5 and S6 Table, cellular process (51 proteins), single-organism process (47 proteins) and biological regulation (43 proteins) were the most represented identified biological processes; the most represented molecular functions categories were binding (47 proteins) and catalytic activity (17 proteins); the most represented cellular components categories were the cell compartment (50 proteins), the organelle compartment (49 proteins) and the membrane compartment (35 proteins). Some GO terms’ corresponding differentially expressed proteins’ proportions in their collection are significantly different from the GO term corresponding proteins’ proportions in the collection of all qualitative proteins (S6 Table). The top ten represented GO categories were intermediate filament, extracellular organelle, extracellular vesicular exosome, extracellular membrane-bounded organelle, extracellular region, developmental process, extracellular region part, the receptor) for advanced glycation endproducts (RAGE) receptor binding, keratin filament and tissue development (Fig 6).

### KEGG pathway annotation

**Fig 7:**
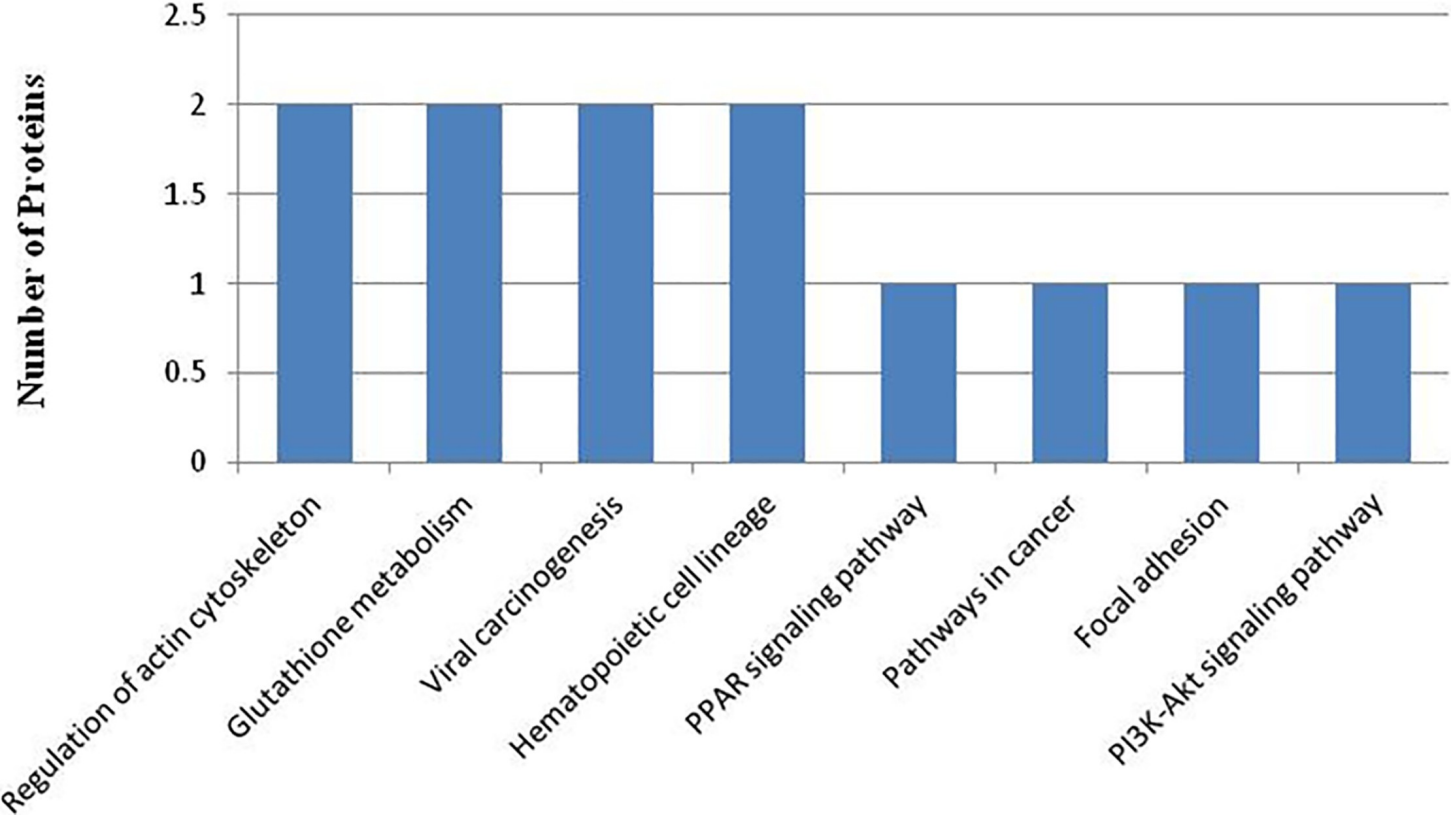
The most represented KEGG pathways.

**Fig 8:**
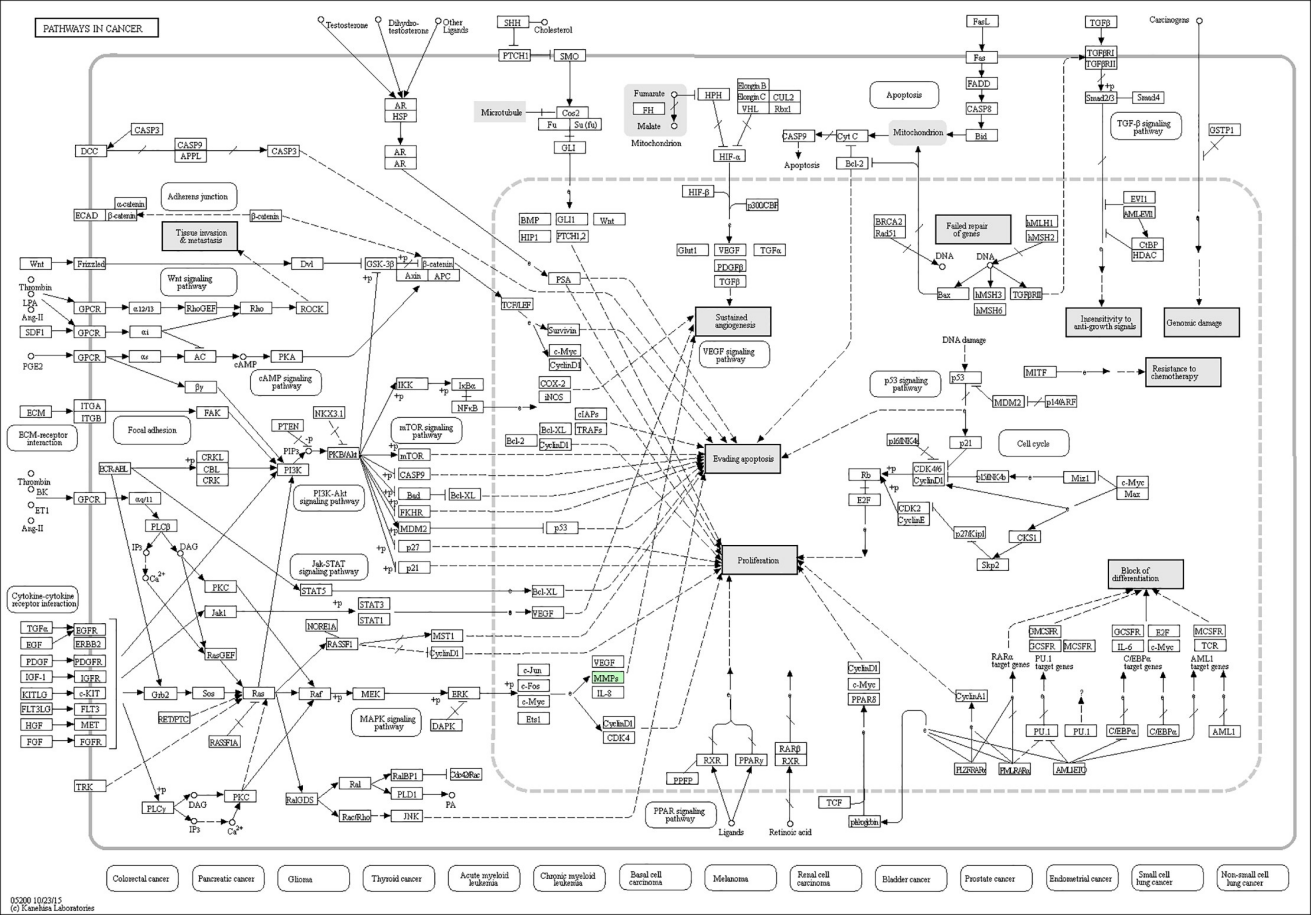
Representative KEGG pathway for pathways in cancer, as per the citation guidelines: www.kegg.jp/kegg/kegg1.html. The differentially expressed proteins were mapped as green [17–19].

**Fig 9:**
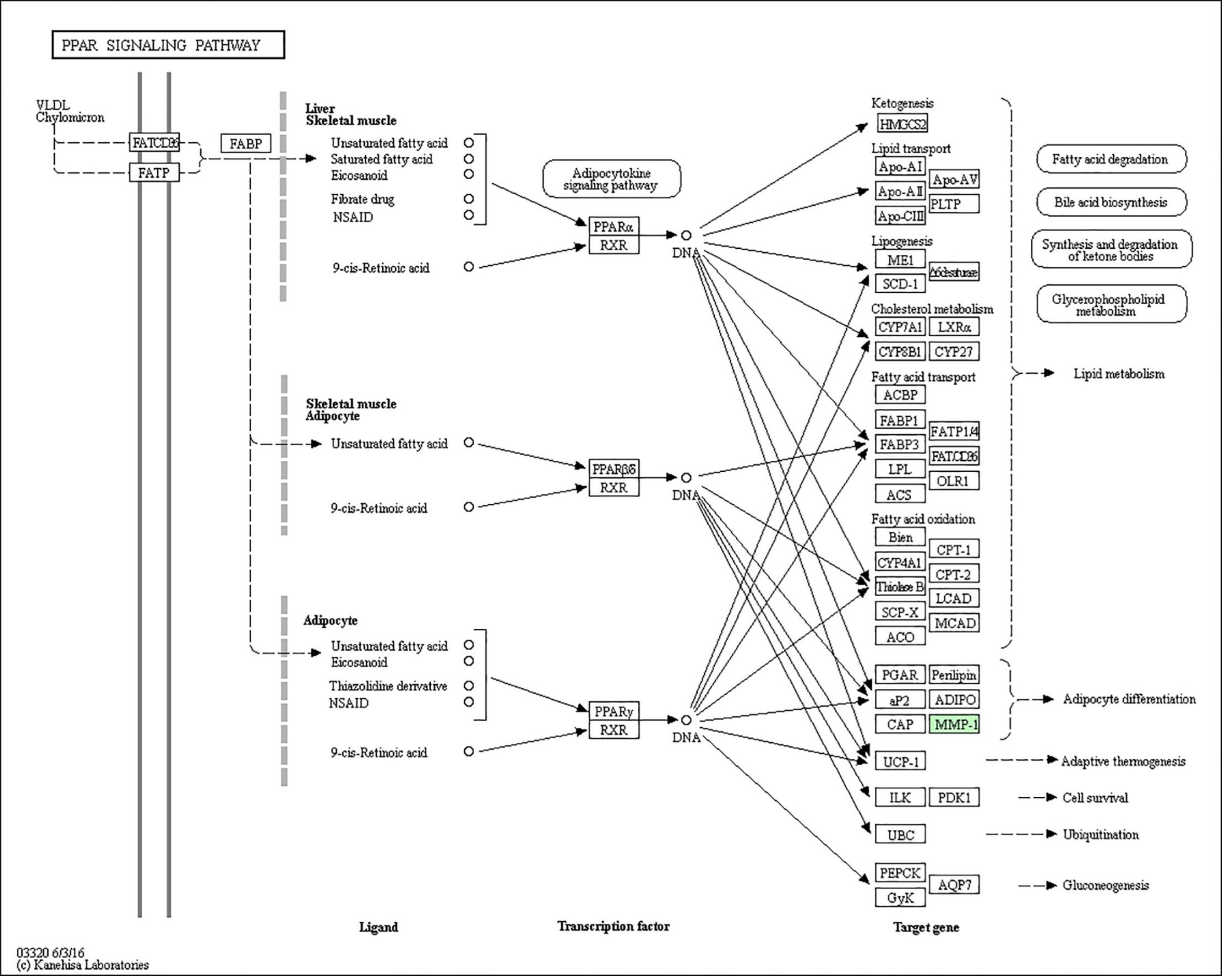
Representative KEGG pathway for PPAR signaling pathway, as per the citation guidelines: www.kegg.jp/kegg/kegg1.html. The differentially expressed proteins were mapped as green [17–19].

**Fig 10:**
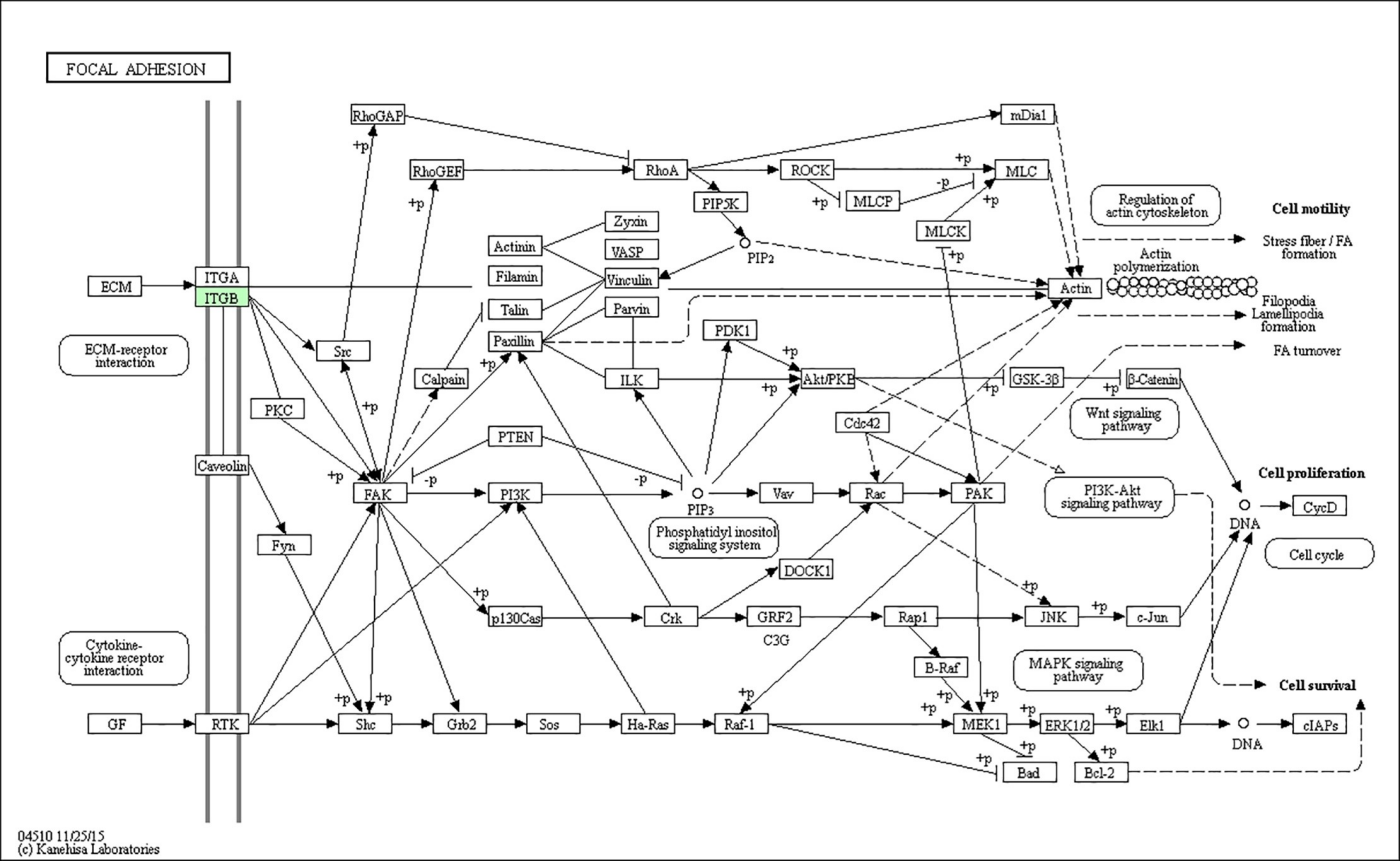
Representative KEGG pathway for focal adhesion, as per the citation guidelines: www.kegg.jp/kegg/kegg1.html. The differentially expressed proteins were mapped as green [17–19].

A protein need to interact and cooperate with other proteins to complete a biochemical reaction in biological systems, so a KEGG pathway that is a collection of manually drawn pathway maps representing our knowledge on the molecular interaction and reaction networks based analysis [14] was performed to identify biochemical pathways that would be potentially affected by proteins differentially expressed in PC-3M-1E8 cells compared with PC-3M-2B4 cells. We established pathway associations for 58 differential proteins with 47 unique KEGG maps. Two significantly up- or down-regulated proteins in PC-3M-1E8/PC-3M-2B4 comparison group processing in four pathways identified included regulation of actin cytoskeleton, glutathione metabolism, viral carcinogenesis and hematopoietic cell lineage, and other 43 pathways each included one differentially expressed protein (Fig 7 and Table 2). All KEGG analysis results are shown in S7 Table. The proteins with differential abundances involved in pathways in cancer, PPAR signaling pathway and focal adhesion were colored in Figs 8, 9 and 10, respectively.

**Table 2:** KEGG pathway analysis of the differentially expressed proteins (top eight pathways).

Pathway	Associated proteins
Regulation of actin cytoskeleton	Integrin beta, cDNA FLJ55803, highly similar to Gelsolin
Glutathione metabolism	Aminopeptidase N (AP-N), Glutathione S-transferase
Viral carcinogenesis	TATA-box-binding protein (TBP), cDNA FLJ55803, highly similar to Gelsolin
Hematopoietic cell lineage	Aminopeptidase N (AP-N), cDNA FLJ51032, highly similar to CD9 antigen
Pathways in cancer	MMP1 protein
PPAR signaling pathway	MMP1 protein
Focal adhesion	Integrin beta
PI3K-Akt signaling pathway	Integrin beta

### Protein-protein interaction (PPI) analysis

**Fig 11:**
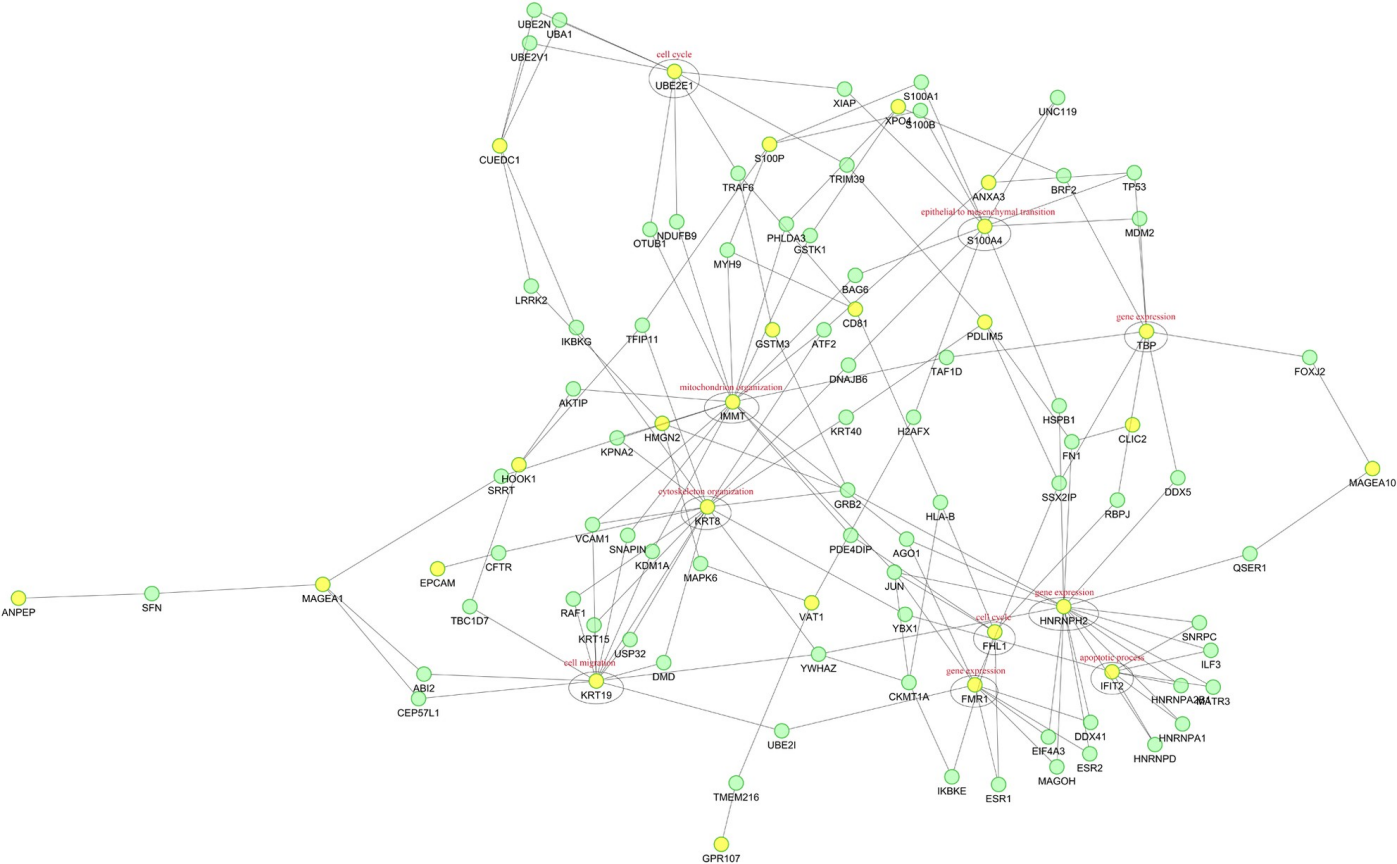
Potential protein-protein interaction networks. The nodes: the proteins; the lines between the nodes: protein-protein interaction modes; yellow spots: proteins that expressed significantly differentially; green spots: proteins that did not express differentially.

To predict the relationship among all these identified differentially expressed proteins in PC-3M-1E8/PC-3M-2B4 cells, a PPI network was constructed using STRING Database version 10.0. As shown in Fig 11, 26 differentially expressed functionally linked proteins in PC-3M-1E8/PC-3M-2B4 comparison group were mainly enriched in the term “gene expression”, “mitochondrion organization”, “cytoskeleton organization”, “cell migration”, “epithelial to mesenchymal transition”, “cell cycle” and “apoptotic process”. The most linked proteins were HNRNPH2, IMMT, KRT8, KRT19, S100A4, FMR1, UBE2E1, TBP, IFIT2, FHL1, etc. KRT8 and KRT19 were identified as directly interacting proteins, whereas other proteins’ interactions identified were indirect. The enrichment of “cytoskeleton organization”, “cell migration” and “epithelial to mesenchymal transition” were consistent with the highly metastatic potential of PC-3M-1E8 cells.

### Validation of differentially expressed proteins by qRT-PCR and western blot analysis

**Fig 12:**
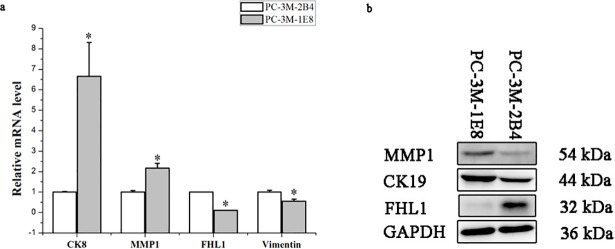
Validation of selected candidate proteins identified from iTRAQ expression in PC-3M-1E8 and PC-3M-2B4 cells with qRT-PCR and western blot analysis. a. qRT-PCR: The relative transcription levels of MMP1 and CK8 significantly increased, but the relative transcription levels of vimentin and FHL1 significantly decreased in the PC-3M-1E8 cells compared with the PC-3M-2B4 cells. GAPCH was used as an internal reference. The data represent mean ± SD of three biological replicates. *P < 0.05. b. Western blot: The expression level of MMP1 and CK19 significantly increased, but the protein level of FHL1 significantly decreased in the PC-3M-1E8 cells compared with the PC-3M-2B4 cells. GAPDH was performed as internal reference. Full-length gels are presented in S2 Fig. Experiments were repeated three times independently.

On the basis of the proteomic analysis results, five important iTRAQ-identified proteins (CK8, CK19, MMP1, vimentin and FHL1) were selected for further validation of their expression patterns in the PC-3M-1E8 and PC-3M-2B4 groups by western blot. GAPDH was employed as an internal reference to confirm that the total protein loading quantity of different samples is identical. A significant increase in CK8, CK19 and MMP1 expression and a marked decrease in vimentin and FHL1 expression were observed in PC-3M-1E8 cells as compared with PC-3M-2B4 cells (Figs 1 and 12B), which were consistent with those derived from the iTRAQ studies (S5 Table). Four of them (CK8, MMP1, vimentin and FHL1) were confirmed by qRT-PCR (Fig 12A).

### Knock down of MMP1 suppresses *in vitro* migration and invasion of the PC-3M-1E8 cells

**Fig 13:**
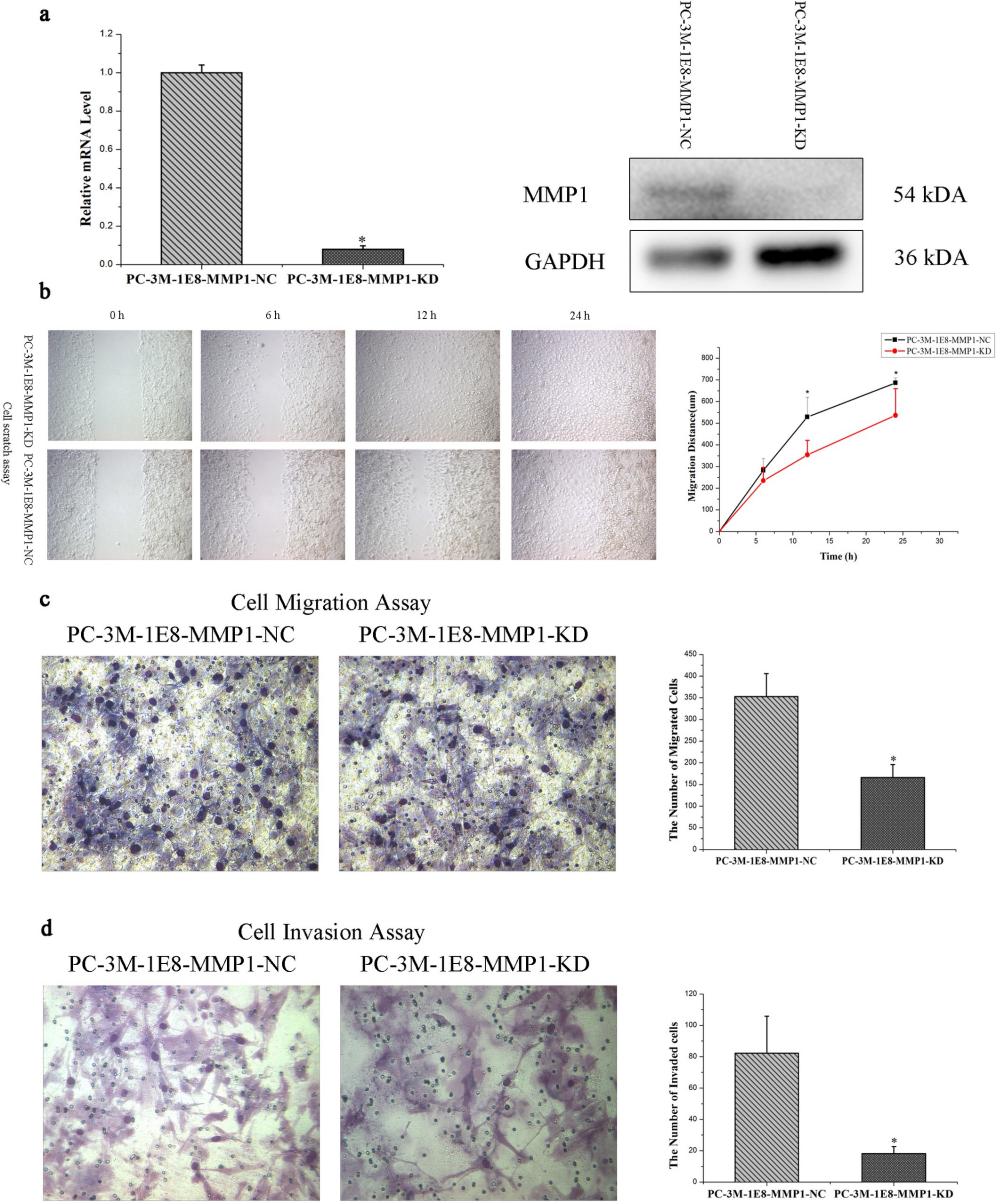
Knock down of MMP1 suppresses *in vitro* migration and invasion of the PC-3M-1E8 cells. (a) Left panel: the PC-3M-1E8-MMP1-KD cells stably knocked down MMP1 by MMP1 siRNA stable transfection were identified and confirmed using qRT-PCR analysis. The relative transcription levels of MMP1 significantly decreased in the PC-3M-1E8-MMP1-KD cells compared with the PC-3M-1E8-MMP1-NC cells. GAPCH was used as an internal reference. The data represent mean ± SD of three biological replicates. *P < 0.05. Right panel: the PC-3M-1E8-MMP1-KD cells stably knocked down MMP1 by MMP1 siRNA stable transfection were identified and confirmed using western blot analysis. The expression of MMP1 significantly decreased in the PC-3M-1E8-MMP1-KD cells compared with the PC-3M-1E8-MMP1-NC cells. GAPDH was performed as internal reference. Full-length gels are presented in S3 Fig. Experiments were repeated three times independently. (b-d) Effects of MMP1 siRNA stable transfection on the *in vitro* migratory and invasive potential of PC-3M-1E8 cells. The *in vitro* migratory (b: cell scratch assay, c: cell migration assay) and invasive (d: cell invasion assay) potential significantly decreased in the PC-3M-1E8-MMP1-KD cells compared with the PC-3M-1E8-MMP1-NC cells. The data represents mean ± SD of 3 repeats. Significantly different according to Student’s t-test, *P<0.05.

To further evaluate the role of MMP1 overexpression in regulation of the metastatic ability of the PC-3M-1E8 cells, PC-3M-1E8 cells were stably transfected with a siRNA against MMP1 that dramatically knocked down the expression of MMP1 (PC-3M-1E8-MMP1-KD) as compared with the negative control stably transfected with the non-targeting sequence (PC-3M-1E8-MMP1-NC), as detected by qRT-PCR and western blot of the transfected cells (Fig 13A). Our results demonstrated that PC-3M-1E8-MMP1-KD cells showed statistically significantly slower in scratch closure than PC-3M-1E8-MMP1-NC cells in a cell scratch assay (Fig 13B), in addition, fewer cells that migrated and invaded across 8 μm diameter pores over 48 hours were present in the siMMP1-transfected group (PC-3M-1E8-MMP1-KD) than in the negative control group (PC-3M-1E8-MMP1-NC) (Fig 13C and 13D), revealing that MMP1 silencing remarkably weakened the cellular *in vitro* migratory and invasive capabilities of the PC-3M-1E8 cells. These results confirm that MMP1 is a functional protein associated with the higher metastatic ability of PC-3M-1E8 cells than PC-3M-2B4 cells.

## Discussion

Tumor metastases are the main cause of death and pain in clinically advanced prostate cancer. Increased understanding of the initial molecular mechanisms regulating the largely obscure PCa metastasis will increase the possibilities for identifcation of new tumor biomarkers or more efficient intervention strategies to prevent or treat this deadly disease in the future. As indispensable complementarity to most proteomics analysis of patients’ samples that don’t allow for any manipulation of the system, *in vitro* cell lines, like PC-3M-1E8 and PC-3M-2B4 cell lines, closely mimicing the clinical condition in patients in which each parameter can be varied in a logical manner are crucial to investigate the molecular pathogenesis of prostate cancer and develop potent novel therapeutic agents. The fact that PC-3M-1E8 and PC-3M-2B4 cell lines derived by limiting dilution cloning from the same PC-3M cell line have different metastatic ability suggests heterogeneity in the original cell line [8]. Indeed, cell culture may generate cellular heterogeneity [20], this is in accord with tumor heterogeneity [21]. As two sublines of PC-3M cell line but with different metastatic potential, PC-3M-1E8 and PC-3M-2B4 cell lines provide an invaluable resource to dissect the molecular mechanisms responsible for prostate cancer metastasis and study new therapeutic strategies targeting different metastatic ability of cancer cells both *in vitro* and *in vivo* [22–24].

Our data verified the characterization of PC-3M-1E8 cells as a highly metastatic subline of PC-3M cells and PC-3M-2B4 as a poorly metastatic subline of PC-3M cells. The all 19 highly polymorphic STR markers and Amelogenin locus in alleles 1 and 2 in PC-3M-1E8 and PC-3M-2B4 cells showed 100% homology with PC-3M cells. This data authenticated that PC-3M-1E8 and PC-3M-2B4 cell lines are lineage derivations from PC-3M cells [8]. PC-3M-1E8 cell proliferation was not statistically different from the PC-3M-2B4 cells (data not shown). Like their parental cell counterpart PC-3M cells, they didn’t express prostate-specific marker PSA (data not shown). In addition, both PC-3M-1E8 and PC-3M-2B4 were concomitantly positive for the luminal epithelial marker CK8 but lack of expression of the basal cell marker CK5, ascertaining the luminal epithelial origin of them, although PC-3M-1E8 cells exhibited strong staining but PC-3M-2B4 cells exhibited low labelling for CK8. In contrast to PC-3M-2B4 cells, PC-3M-1E8 cells expressed substantially lower levels of fibroblast (vimentin) associated marker, but stromal (HGFα) associated marker was constitutively activated in both of them [25]. The *in vitro* cell migration, invasion, and scratch assays indicated that the cellular migratory, invasive, and motile abilities of PC-3M-1E8 cells were stronger than that of PC-3M-2B4 cells, which confirmed PC-3M-1E8 cells have significantly greater metastatic ability *in vitro* than PC-3M-2B4 cells [8, 26].

In this study, a comprehensive iTRAQ-based quantitative proteomic analysis that is a robust protein discovery technique was performed and validated by qRT-PCR and western blot analysis to assess the total proteome of PC-3M-1E8 and PC-3M-2B4 cells and to identify the biological process and pathways responsible for their different metastatic ability. Here, a total of 58 proteins were shown to be significantly differentially expressed between PC-3M-1E8 and PC-3M-2B4 cells, then they were used to perform GO functional annotations, KEGG intracellular pathway annotation and PPI analysis, which help us comprehensively analyze the differentially expressed proteins. All the 58 significantly differentially expressed proteins were annotated to 817 GO terms and participated in 47 unique KEGG pathways, suggesting besides changes in metastatic ability, several other specific cellular pathways alterations like glutathione metabolism and viral carcinogenesis, etc., also exist between PC-3M-1E8 and PC-3M-2B4 cells, which may help us do research to illuminate their characteristics and enlarge their application in additional areas in the future. To validate the data, we subsequently measured protein expression pattern of CK8, CK19, MMP1, vimentin and FHL1 using western blot analysis and four of them (CK8, MMP1, vimentin and FHL1) were confirmed by qRT-PCR, which showed a similar pattern to the observations found in the iTRAQ studies. The discrepancy in CK19 expression between mRNA and protein level in PC-3M-1E8/PC-3M-2B4 cells might result from the dysregulation of post-transcriptional modification and degradation mechanisms in these two cell lines and merits further investigation. This pattern or profile of protein expression in these two cell lines was not consistent with a previous literature that maybe as a result of different cell passages, experimental methods or conditions used in these two studies, so the importance of other proteins in high metastatic ability of PC-3M-1E8 cells should not be underestimated [26]. In addition, functional analyses indicated that silencing one selected differentially expressed protein MMP1 remarkably weakened the cellular *in vitro* migratory and invasive capabilities of the PC-3M-1E8 cells, which demonstrated that MMP1 is a positive regulator of higher metastatic ability of PC-3M-1E8 cells than PC-3M-2B4 cells.

Epithelial-mesenchymal transition (EMT), which plays an important role in tumor migration, invasion and metastasis, is often characterized by loss of E-cadherin at cell-cell junctions and increased expression of mesenchymal-associated genes such as vimentin [27, 28]. However, the level of vimentin was not higher in PC-3M-1E8 cells and even lower than in PC-3M-2B4 cells, indicating that EMT may not be the main reason for the increased metastatic ability of PC-3M-1E8 cells and that it has not undergone extensive EMT, even though stromal cells associated marker HGFα was constitutively activated in both of them, suggesting they have experienced a certain degree of EMT, which is supported by its higher expression of CK8 and CK19 than PC-3M-2B4 cells and tightly packed polyhedral cobblestone growth pattern characteristic of epithelial cells, but PC-3M-2B4 cells adopted a spindle-shaped morphology (data not shown). As keratin-containing intermediate filament proteins, cytokeratins (CKs) form important structural components of epithelial cytoskeleton [29]. CK19, which is an acidic keratin with lowest molecular weight (40 kDa) among the 20 CKs, is part of the cytoskeletal structure of simple epithelia and their malignant counterparts [29].

Interestingly, MMP1 expression level was significantly higher in PC-3M-1E8 cells than in PC-3M-2B4 cells. This was consistent with that mRNA levels of MMP1 in more aggressive PC3 cells was significantly higher than that of non-invasive LNCaP cells under basal conditions [30]. *In vitro* functional analyses indicated that silencing MMP1 remarkably weakened the cellular *in vitro* migratory and invasive capabilities of the PC-3M-1E8 cells, which demonstrated that the elevated level of MMP1 in PC-3M-1E8 cells may be one of the mechanisms of higher metastatic ability of these cells. Matrix metalloproteinases (MMPs) that degrade structural components of the basement membrane and extracellular matrix are a critical family of metal dependent proteolytic enzymes, thereby have a key role in tumor migration, invasion and metastasis [31]. MMPs are also contribute to several other processes of cancer development: regulating cancer cell growth and apoptosis, differentiation, apoptosis, angiogenesis and immune responses to cancer [31]. MMP1 is a critical collagenase regulating cancer cells migration and metastasis and its major substrates are collagens I, II and III [31]. MMP1 expression was significantly higher in prostate carcinoma versus in benign prostatic hypertrophy [32–34] and in high- (Gleason scores 8, 9) and intermediate- (Gleason score 7) grade versus in low-grade (Gleason scores 4, 5, 6) prostate carcinomas [35]. Further, overexpression of MMP1 in prostate cancer cells induced cells migration and invasion *in vitro* and the incidence of lung metastasis *in vivo*, but blocking MMP1 function significantly inhibited prostate cancer cell migration, invasion and metastasis [34, 36]. It is suggested that MMP1 can become a future target for prostate cancer metastasis therapy and PC-3M-1E8 cell line may be a suitable model for testing MMP1-based therapeutics.

Furthermore, PC-3M-1E8 cells showed a significant down-regulated expression of FHL1 compared with PC-3M-2B4 cells. FHL1 is significantly down-regulated expressed and plays important roles in the regulation of development and progression in several types of human tumors including prostatic carcinoma, bladder cancer (BC), breast carcinoma, hepatocarcinoma, renal carcinoma, lung cancer, gastric cancer, astrocytoma, oral squamous cell carcinoma (OSCC), and melanoma [37–41]. Increased the expression of FHL1 could remarkably block anchorage-dependent and -independent cell growth, migration, invasion, and distant metastasis [38–40, 42]. The decreased expression of FHL1 in the PC-3M-1E8 cell line may contribute to promotion of cell migration and invasiveness. *FHL1* is a tumor suppressor gene that acts downstream of the Src tyrosine kinase and the focal adhesion adaptor protein Crk-associated substrate (Cas) to specifically block anchorage-independent cell growth and migration [37]. The mechanism of FHL1 down-regulation in gastrointestinal cancers, BC and OSCCs is through DNA methylation of the promoter region [39, 41, 42]. The down-regulation of FHL1, which specifically combined to the activation function domain-1 of oestrogen receptor (ER) involving in the development and progression of breast cancer, caused ER to be recruited to an estrogen-responsive promoter and bind to an estrogen-responsive element and repressed the transcription and translation of estrogen-responsive genes [43, 44]. FHL1 acts in activation of the tumor suppressor gene *p21* and repression of the oncogene *c-myc* through physically and functionally interacting with Smad2, Smad3 and Smad4 that are important regulators of cancer development and progression in a casein kinase 1δ-dependent manner [45].

In conclusion, a comprehensive proteomic approach based on iTRAQ was applied to analyze the differentially expressed proteins of PC-3M-1E8 and PC-3M-2B4 cells which identified 58 differentially expressed proteins. The bioinformatic analysis suggests that the differentially expressed proteins, like MMP1 and FHL1, may contribute to the higher metastatic potential of PC-3M-1E8 cells than PC-3M-2B4 cells. In addition, functional analyses proved MMP1’s effect on the higher metastatic ability of PC-3M-1E8 cells than PC-3M-2B4 cells. These findings provided a unique resource to specifically reveal the complex molecular regulatory mechanisms underlying the progression of prostate cancer from poorly-metastatic to highly-metastatic stage. Because cell lines accumulated changes in their phenotype (including protein expression) after being maintained in artificial condition for several generations, the findings of the present study may not be appropriate to be applied to *in vivo* PCa tissue directly. Further studies are necessary to elucidate the comprehensive molecular mechanisms of the metastatic potential differences of these two homologous cell lines and address their functions on metastasis of PCa.

## Supporting information

S1 Fig Full-length gels of blots in Fig 1. (DOC)

S2 Fig Full-length gels of blots in Fig 12. (DOC)

S3 Fig Full-length gels of blots in Fig 13. (DOC)

S1 Table Details for the primary antibodies used for western blot analysis. (DOC)

S2 Table Details for the primer sequences used for qRT-PCR. (DOC)

S3 Table Total peptides identified from PC-3M-1E8 cells and PC-3M-2B4 cells using LC-MS/MS analysis. (XLSX)

S4 Table Total proteins identified from PC-3M-1E8 cells and PC-3M-2B4 cells using LC-MS/MS analysis. (XLSX)

S5 Table Differential abundant proteins identified from PC-3M-1E8 cells and PC-3M-2B4 cells using iTRAQ analysis. (XLSX)

S6 Table GO annotation of the differentially abundant proteins from PC-3M-1E8 cells and PC-3M-2B4 cells. (XLSX)

S7 Table KEGG pathway annotation of the differentially abundant proteins from PC-3M-1E8 cells and PC-3M-2B4 cells. (XLSX)
