# Supplementary material for: Proteomic analysis of human prostate cancer PC-3M-1E8 cells and PC-3M-2B4 cells of same origin but with different metastatic potential
Source: PLoS One. 2018 Oct 31;13(10):e0206139. doi: 10.1371/journal.pone.0206139 (PMC6209233; doi:10.1371/journal.pone.0206139)
Supplement: S1 Fig — (DOC) [file pone.0206139.s001.doc]

**Proteomic Analysis of Human Prostate Cancer**

**PC-3M-1E8 cells and PC-3M-2B4 cells of Same Origin**

**but with Different Metastatic Potential**

Shujiang Zhang, Chengcheng Zheng, Shunheng Yao, Zhonghui Wang, Li Xu, Rongfu Yang, Xiang Meng, Jianhui Wu, Li Zhou, Zuyue Sun


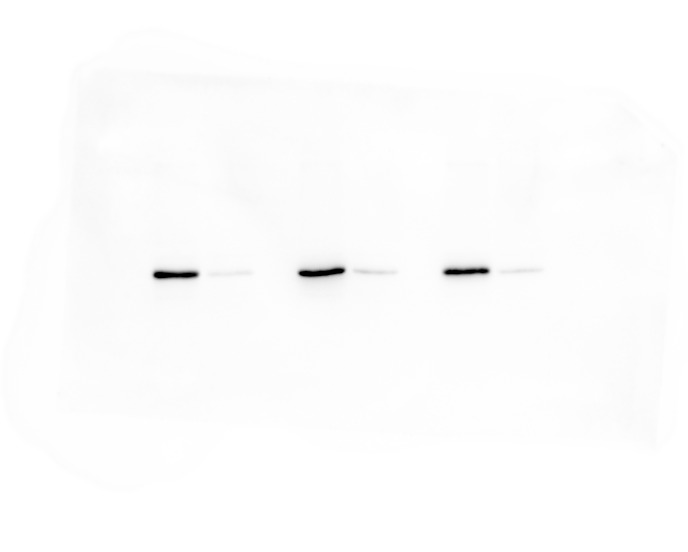


CK8


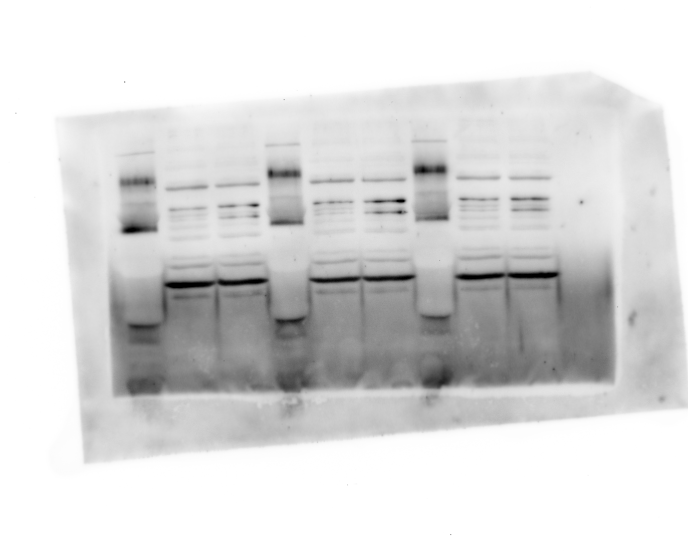


HGFα


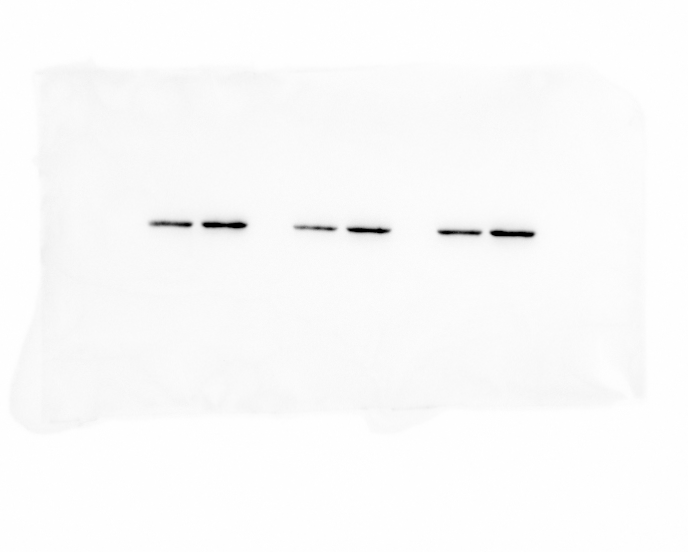


Vimentin


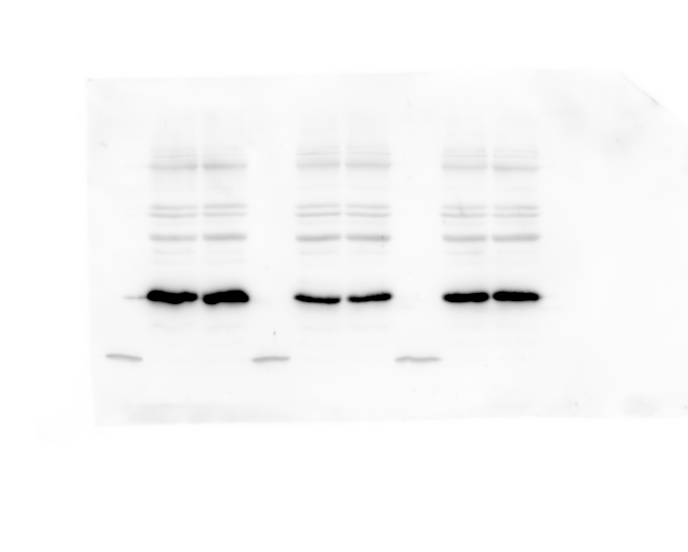


GAPDH

S1 Figure. Full-length gels of blots in Figure 1. Expression of cytodifferentiation and prostate-specific markers in PC-3M-1E8 and PC-3M-2B4 cells. Western blot analysis was performed on whole cell lysate using antibodies against epithelial (CK8), stromal (HGFα), and fibroblast (vimentin) markers. GAPDH was used as protein loading control.

PC-3M-2B4

PC-3M-1E8

PC-3M-2B4

PC-3M-1E8

PC-3M-2B4

PC-3M-1E8
