# Supplementary material for: Proteomic analysis of human prostate cancer PC-3M-1E8 cells and PC-3M-2B4 cells of same origin but with different metastatic potential
Source: PLoS One. 2018 Oct 31;13(10):e0206139. doi: 10.1371/journal.pone.0206139 (PMC6209233; doi:10.1371/journal.pone.0206139)

**Proteomic Analysis of Human Prostate Cancer**

**PC-3M-1E8 cells and PC-3M-2B4 cells of Same Origin**

**but with Different Metastatic Potential**

Shujiang Zhang, Chengcheng Zheng, Shunheng Yao, Zhonghui Wang, Li Xu, Rongfu Yang, Xiang Meng, Jianhui Wu, Li Zhou, Zuyue Sun


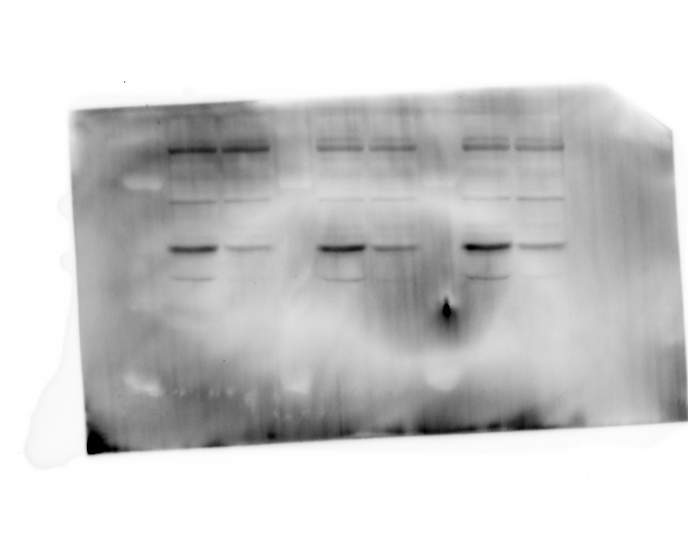


MMP1


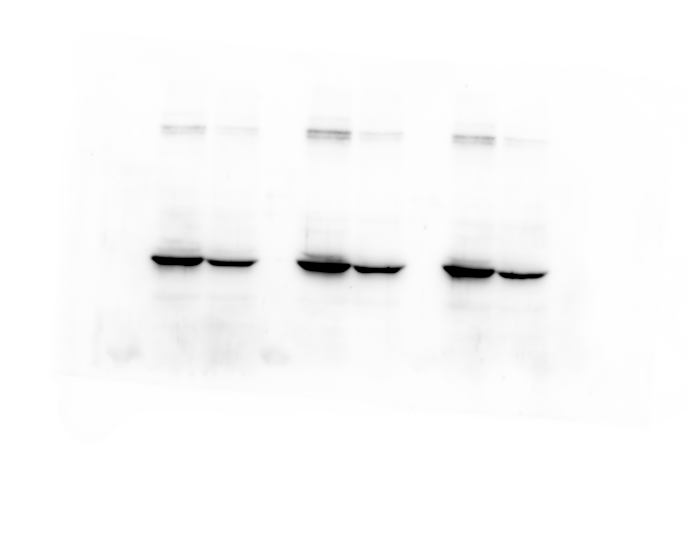


CK19

FHL1


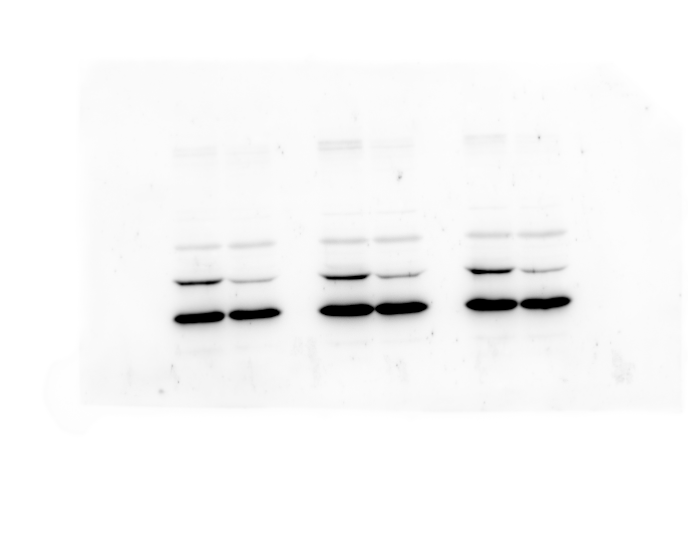


GAPDH

S2 Figure. Full-length gels of blots in Figure 12. Validation of selected candidate proteins identified from iTRAQ expression in PC-3M-1E8 and PC-3M-2B4 cells with western blot analysis. Western blot: The expression level of MMP1 and CK19 significantly increased, but the protein level of FHL1 significantly decreased in the PC-3M-1E8 cells compared with the PC-3M-2B4 cells. GAPDH was performed as internal reference. Experiments were repeated three times independently.

PC-3M-2B4

PC-3M-1E8

PC-3M-2B4

PC-3M-1E8

PC-3M-2B4

PC-3M-1E8


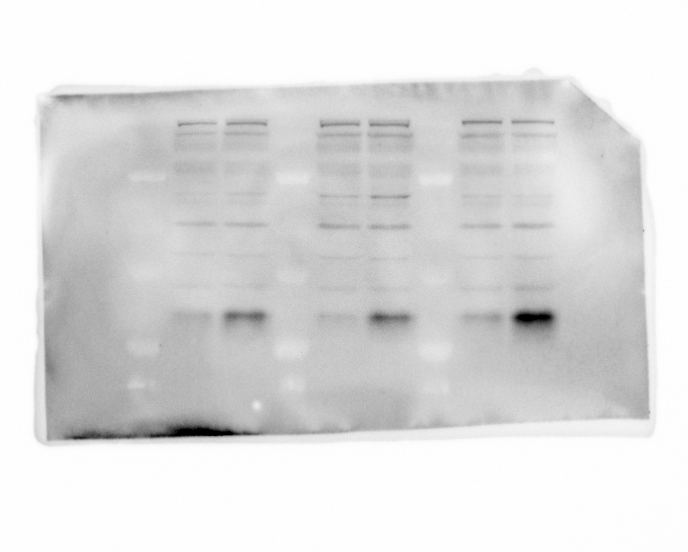

Supplement: S2 Fig — (DOC) [file pone.0206139.s002.doc]
