# Supplementary material for: Proteomic analysis of human prostate cancer PC-3M-1E8 cells and PC-3M-2B4 cells of same origin but with different metastatic potential
Source: PLoS One. 2018 Oct 31;13(10):e0206139. doi: 10.1371/journal.pone.0206139 (PMC6209233; doi:10.1371/journal.pone.0206139)
Supplement: S3 Fig — (DOC) [file pone.0206139.s003.doc]

**Proteomic Analysis of Human Prostate Cancer**

**PC-3M-1E8 cells and PC-3M-2B4 cells of Same Origin**

**but with Different Metastatic Potential**

Shujiang Zhang, Chengcheng Zheng, Shunheng Yao, Zhonghui Wang, Li Xu, Rongfu Yang, Xiang Meng, Jianhui Wu, Li Zhou, Zuyue Sun


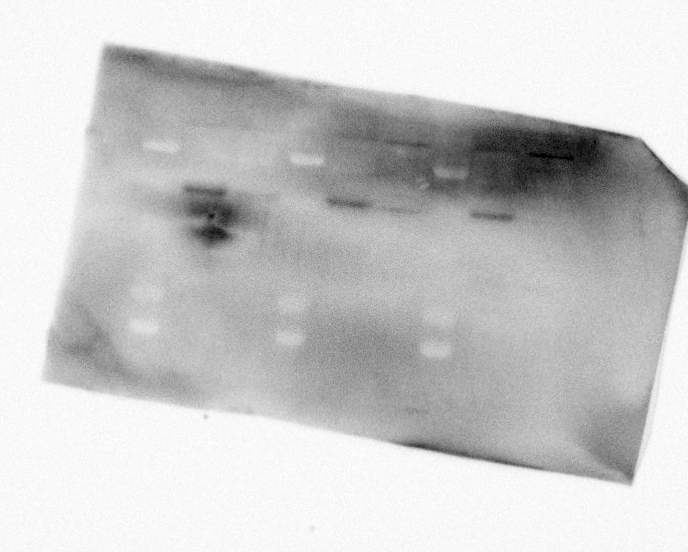

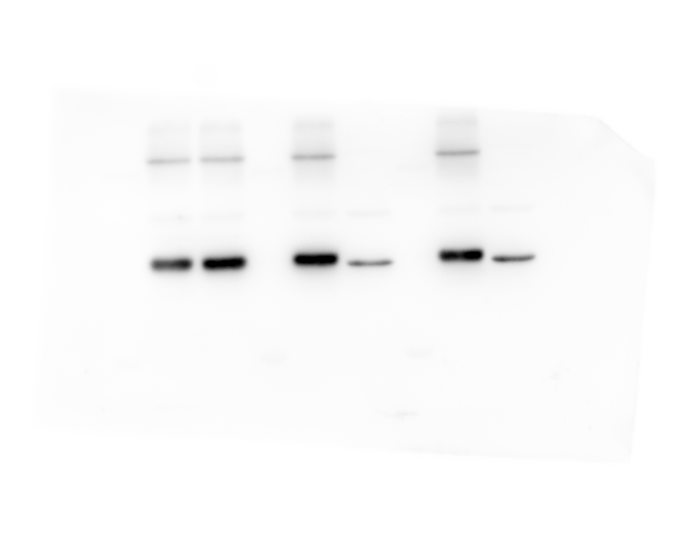


GAPDH

MMP1

S3 Figure. Full-length gels of blots in Figure 13. The PC-3M-1E8-MMP1-KD cells stably knocked down MMP1 by MMP1 siRNA stable transfection were identified and confirmed using western blot analysis. The expression of MMP1 significantly decreased in the PC-3M-1E8-MMP1-KD cells compared with the PC-3M-1E8-MMP1-NC cells. GAPDH was performed as internal reference. Experiments were repeated three times independently.

PC-3M-1E8-MMP1-KD

PC-3M-1E8-MMP1-NC

PC-3M-1E8-MMP1-KD

PC-3M-1E8-MMP1-NC

PC-3M-1E8-MMP1-KD

PC-3M-1E8-MMP1-NC
