# Supplementary material for: Proteomic analysis of human prostate cancer PC-3M-1E8 cells and PC-3M-2B4 cells of same origin but with different metastatic potential
Source: PLoS One. 2018 Oct 31;13(10):e0206139. doi: 10.1371/journal.pone.0206139 (PMC6209233; doi:10.1371/journal.pone.0206139)
Supplement: S1 Table — (DOC) [file pone.0206139.s004.doc]

**Proteomic Analysis of Human Prostate Cancer**

**PC-3M-1E8 cells and PC-3M-2B4 cells of Same Origin**

**but with Different Metastatic Potential**

Shujiang Zhang, Chengcheng Zheng, Shunheng Yao, Zhonghui Wang, Li Xu, Rongfu Yang, Xiang Meng, Jianhui Wu, Li Zhou, Zuyue Sun

**Supplementary Table S1. Details for the primary antibodies used for western blot analysis**

| **Antibody** | **Source** | **Cat #** | **Dilutions** |
| --- | --- | --- | --- |
| PSA | [Cell Signaling Technology](http://www.baidu.com/link?url=sr3WYGgE6DLCtvFX_18haA3A1rOb8c7V84MPma1x6LQRs7sCqCdtkHzAZIJYapSi) | 2475 | 1: 1000 |
| PSMA | Santa Cruz | sc-59674 | 1: 200 |
| CK8 | Santa Cruz | sc-130312 | 1: 200 |
| CK5 | Santa Cruz | sc-32721 | 1: 200 |
| HGFα | Santa Cruz | sc-7949 | 1: 200 |
| Vimentin | Santa Cruz | sc-6260 | 1: 1000 |
| FHL-1 | Santa Cruz | sc-374246 | 1: 100 |
| MMP1 | Abcam | ab134184 | 1: 1000 |
| CK19 | Abcam | ab52625 | 1: 4500 |
| GAPDH | [Cell Signaling Technology](http://www.baidu.com/link?url=sr3WYGgE6DLCtvFX_18haA3A1rOb8c7V84MPma1x6LQRs7sCqCdtkHzAZIJYapSi) | 5174 | 1: 1000 |
