# Supplementary material for: Proteomic analysis of human prostate cancer PC-3M-1E8 cells and PC-3M-2B4 cells of same origin but with different metastatic potential
Source: PLoS One. 2018 Oct 31;13(10):e0206139. doi: 10.1371/journal.pone.0206139 (PMC6209233; doi:10.1371/journal.pone.0206139)
Supplement: S2 Table — (DOC) [file pone.0206139.s005.doc]

**Proteomic Analysis of Human Prostate Cancer**

**PC-3M-1E8 cells and PC-3M-2B4 cells of Same Origin**

**but with Different Metastatic Potential**

Shujiang Zhang, Chengcheng Zheng, Shunheng Yao, Zhonghui Wang, Li Xu, Rongfu Yang, Xiang Meng, Jianhui Wu, Li Zhou, Zuyue Sun

| Primer | Sequence |
| --- | --- |
| MMP1/FORWARD | AGAGCAGATGTGGACCATGC |
| MMP1/REVERSE | TTGTCCCGATGATCTCCCCT |
| FHL1/FORWARD | TGGCACAAAGACTGCTTCAC |
| FHL1/REVERSE | AATGCTTGGCAAACTTGGTC |
| KRT8/FORWARD | CGAGATCGCCACCTACAGGA |
| KRT8/REVERSE | CATAGCCGCTGGTGGTCTTC |
| KRT19/FORWARD | GATAGTGAGCGGCAGAATCA |
| KRT19/REVERSE | GACCTTGGAGGCAGACAAAT |
| VIM/FORWARD | GGACCAGCTAACCAACGACA |
| VIM/REVERSE | AAGGTCAAGACGTGCCAGAG |
| GAPDH/FORWARD | TGACTTCAACAGCGACACCCA |
| GAPDH/REVERSE | CACCCTGTTGCTGTAGCCAAA |

**Supplementary Table S2. Details for the primer sequences used for qRT-PCR**
